# Molecular Pathobiology of the Cerebrovasculature in Aging and in Alzheimers Disease Cases With Cerebral Amyloid Angiopathy

**DOI:** 10.3389/fnagi.2021.658605

**Published:** 2021-05-17

**Authors:** Joseph O. Ojo, Jon M. Reed, Gogce Crynen, Prashanthi Vallabhaneni, James Evans, Benjamin Shackleton, Maximillian Eisenbaum, Charis Ringland, Anastasia Edsell, Michael Mullan, Fiona Crawford, Corbin Bachmeier

**Affiliations:** ^1^Roskamp Institute, Sarasota, FL, United States; ^2^James A. Haley Veterans' Hospital, Tampa, FL, United States; ^3^The Open University, Milton Keynes, United Kingdom; ^4^Boehringer Ingelheim Pharmaceuticals, Inc., Ridgefield, CT, United States; ^5^Bay Pines VA Healthcare System, Bay Pines, FL, United States

**Keywords:** cerebrovasculature, Alzheimers disease, cerebral amyloid angiopathy, endothelial cells, mural cells, proteomics, mass spectrometry, perivascular cells

## Abstract

Cerebrovascular dysfunction and cerebral amyloid angiopathy (CAA) are hallmark features of Alzheimer's disease (AD). Molecular damage to cerebrovessels in AD may result in alterations in vascular clearance mechanisms leading to amyloid deposition around blood vessels and diminished neurovascular-coupling. The sequelae of molecular events leading to these early pathogenic changes remains elusive. To address this, we conducted a comprehensive in-depth molecular characterization of the proteomic changes in enriched cerebrovessel fractions isolated from the inferior frontal gyrus of autopsy AD cases with low (85.5 ± 2.9 yrs) vs. high (81 ± 4.4 yrs) CAA score, aged-matched control (87.4 ± 1.5 yrs) and young healthy control (47 ± 3.3 yrs) cases. We employed a 10-plex tandem isobaric mass tag approach in combination with our ultra-high pressure liquid chromatography MS/MS (Q-Exactive) method. Enriched cerebrovascular fractions showed very high expression levels of proteins specific to endothelial cells, mural cells (pericytes and smooth muscle cells), and astrocytes. We observed 150 significantly regulated proteins in young vs. aged control cerebrovessels. The top pathways significantly modulated with aging included chemokine, reelin, HIF1α and synaptogenesis signaling pathways. There were 213 proteins significantly regulated in aged-matched control vs. high CAA cerebrovessels. The top three pathways significantly altered from this comparison were oxidative phosphorylation, Sirtuin signaling pathway and TCA cycle II. Comparison between low vs. high CAA cerebrovessels identified 84 significantly regulated proteins. Top three pathways significantly altered between low vs. high CAA cerebrovessels included TCA Cycle II, Oxidative phosphorylation and mitochondrial dysfunction. Notably, high CAA cases included more advanced AD pathology thus cerebrovascular effects may be driven by the severity of amyloid and Tangle pathology. These descriptive proteomic changes provide novel insights to explain the age-related and AD-related cerebrovascular changes contributing to AD pathogenesis. Particularly, disturbances in energy bioenergetics and mitochondrial biology rank among the top AD pathways altered in cerebrovessels. Targeting these failed mechanisms in endothelia and mural cells may provide novel disease modifying targets for developing therapeutic strategies against cerebrovascular deterioration and promoting cerebral perfusion in AD. Our future work will focus on interrogating and validating these novel targets and pathways and their functional significance.

## Introduction

Alzheimer's disease (AD) is a chronic age-related neurodegenerative disorder and the predominant type of dementia, marked by deposits of amyloid plaques and neurofibrillary tangles composed of hyperphosphorylated tau (Holtzman et al., 2012). To date AD remains untreatable, with very few disease modifying therapeutic approaches advancing into human clinical trials. There is thus an urgent need to identify new biological perspectives behind the neurological dysfunction and underlying etiology of AD.

One of the most common pathological features of AD is vascular dysfunction (De La Torre, 2010). Neuroimaging abnormalities have demonstrated early preclinical features such as cerebral perfusion and metabolic deficits (De la Torre and Mussivand, 1993; de la Torre, 2018), and diminished cortical blood flow beginning many years prior to the onset of neurological symptoms (Binnewijzend et al., 2016; Hays et al., 2016). One of the most common vascular associated lesions in AD is cerebral amyloid angiopathy (CAA), typified by the accumulation of Aβ in leptomeninges and along cerebral blood vessels. Seminal findings from the Nun study also reported lacunar infarcts as a prominent vascular lesion that reduces the neuropathological threshold required for defining the staging of AD neuropathology (Snowdon, 1997). Similar large cohort aging studies have likewise confirmed the presence of underlying cerebrovascular abnormalities such as cerebrovascular small vessel disease (CSVD) lesions as a strong predictor of clinical presentation or cognitive deficits in AD patients (Nagy et al., 1997). Moreover, vascular dementia and AD related dementia both share a significant degree of overlap in their clinical and neuropathological profiles (Kalaria and Ballard, 1999; Kalaria, 2003; Erkinjuntti et al., 2004; Custodio et al., 2017).

At autopsy, other vascular associated morphological lesions can be observed in aging and AD brains such as degeneration of arterioles and leptomeningeal vessels (Vinters et al., 1994; Kalaria, 1996; Farkas and Luiten, 2001), capillaries and mural cells (Miyakawa and Kuramoto, 1989; Hashimura et al., 1991; Kimura et al., 1991; Farkas et al., 2000; Østergaard et al., 2013); mitochondria abnormalities and deposition of phagolysosomes and lipofuscin in cerebrovascular cells (Miyakawa and Kuramoto, 1989); degeneration of the blood brain barrier (Deane and Zlokovic, 2007), arteriolar wall thickening or arteriolosclerosis (Neltner et al., 2014; Blevins et al., 2021), propensity for plaque build up in cerebral arteries(Roher et al., 2004; Beach et al., 2007; Yarchoan et al., 2012), immune cell recruitment and influx of blood borne proteins (Itagaki et al., 1988; Rogers et al., 1988; Togo et al., 2002; Grammas et al., 2006; Di Marco et al., 2015; Merlini et al., 2018).

The consequences of these early vascular changes may significantly impact amyloid clearance and ultimately neuronal metabolism and cerebral brain function (Mosconi, 2005; Mawuenyega et al., 2010). Likewise, amyloid deposition on vascular walls can also induce changes contributing to narrowing and weakening of the vascular wall leading to increased cerebral blood pressure (Kalback et al., 2004; Tian et al., 2004). Whether vascular dysfunction is a prelude to AD pathogenesis or a consequence remains elusive (Govindpani et al., 2019). Understanding the molecular response of the cerebrovasculature during the sequelae of normal aging and AD pathogenesis may provide clues as to the etiology of vascular dysfunction in AD.

Advances in omic approaches has enabled interrogation of different brain regions of AD cases across the neuropathological staging of the disease and age matched healthy controls (Johnson et al., 2018, 2020; Bai et al., 2020). Unbiased proteomic analysis is an extremely powerful tool which can provide a very expansive interrogation of the molecular response in neurodegenerative diseases and can lead to identification of pathogenic mechanisms and novel molecular targets for therapeutic exploration (Zhang et al., 2008). However, very few studies have explored the molecular integrity of the cerebrovasculature in aging and AD.

To address this, we will use our state-of-the-art unbiased proteomic (mass spectrometry) based platform to conduct a detailed characterization and assessment of molecular changes in protein expression levels, cellular origin of proteomic changes, molecular pathways and biofunctions significantly altered in the cerebrovasculature isolated from AD patients compared to aged matched control cases. To further understand the contribution of age, we will also interrogate cases from young (40-50's) compared to aged (70's-90's) control cases.

We utilize a novel protein extraction protocol, separating isolated cerebrovascular samples into cytosolic, membrane and nuclear fractions to increase the depth of the protein mining process, and coupled this with a 10-plex tandem isobaric mass tag (TMT) approach for interrogation with an ultra-high pressure liquid chromatography MS/MS (Q-Exactive) method. We detail the unique and common molecular profiles and pathogenic mechanisms driving cerebrovascular changes in the inferior frontal gyrus of young vs. aged brains, and CAA staged AD vs. age-matched control cases.

## Methods

### Human Tissue and Patient Demographics

Frozen human cortex tissue samples (from the inferior frontal gyrus) were provided mainly from Dr. Thomas Beach, Director of the Brain and Body Donation Program at Sun Health Research Institute (Sun City, AZ) in accordance with the institutional bioethics guidelines. Additional samples were requested from the NIH BrainBank repository (University of Maryland and Ican school of medicine, Mount Sinai, NY). The donors and their respective families provided hand written informed consent for autopsy and the subsequent use of brain tissue for research purposes. For this study, no further ethical approval was required as samples were obtained from deceased, de-identified, and consenting individuals. Neuropathological post-mortem diagnosis of AD (i.e., amyloid plaque and Tangle pathology) was determined using the Consortium to Establish a Registry for Alzheimer's Disease (CERAD) diagnostic criteria and the consensus recommendation by the National Institute for Aging/Reagan Institute Working Group. Braak staging was used to characterize the geographic spread of neurofibrillary tangle pathology (NFT). The severity of CAA was performed according to Vonsattel et al. (1991), and the stage of topographical expansion of CAA was assessed as previously described by Thal et al. (2003) based on a four point numerical conversion per region. Global scores for amyloid, tangle and CAA burden from the microscopic lesion densities were calculated based on the sums of the scores from all regions interrogated. A summary of patient demographics, and clinical information of brain donors used in this proteomic study is provided in Table 1.

Table 1List of control and Alzheimer's disease cases, their demographics, clinical background, APOE genotype, neuropathological score, and randomization of samples for Tandem Mass Tag isobaric 10-plex multiplexing.

**Table:** 

**Groups**	**Sex**	**Age (yrs)**	**ApoE**	**Plaque frontal Cx**	**Plaque total**	**ADNC neuritic plaque score**	**Tangle frontal. Cx**	**tangle total**	**ADNC NFT stage**	**Braak score**	**NIA-R**	**CAA score frontal Cx**	**CAA score total**
Low CAA (≤4)	M	78	2/3	1.5	5.5	C3	0	5	B2	IV	Intermediate	0	0
Low CAA (≤4)	M	77	2/3	3	12.5	C3	1.5	10.5	B2	IV	Intermediate	0	1
Low CAA (≤4)	F	91	2/3	2.5	8	C3	0	5	B2	IV	Intermediate	1	1
Low CAA (≤4)	F	96	3/3	3	12.5	C2	1.16	9.66	B3	V	High	0	0
Low CAA (≤4)	F	84	3/3	3	14.5	C3	3	14.75	B3	VI	High	0	2
Low CAA (≤4)	F	86	3/4	3	13.5	C2	1.5	12.5	B3	V	High	1	3
Low CAA (≤4)	F	92	4/4	3	14.5	C3	3	15	B3	VI	High	0	2
Low CAA (≤4)	M	67	3/4	2.5	11	C3	3	14.5	B3	V	High	1	3
Low CAA (≤4)	F	88	3/4	2.5	11.75	C2	1	9	B2	IV	Intermediate	1	4
Low CAA (≤4)	F	96	3/3	3	13.75	C2	0.5	8	B2	IV	Intermediate	1	
High CAA (≥8)	M	96	2/3	0.5	4	C3	0	7	B2	IV	Intermediate	3	10
High CAA (≥8)	M	89	2/3	3	12.5	C3	1.5	10	B3	V	High	2	8
High CAA (≥8)	F	88	4/4	2	13	C3	2	13	B3	VI	High	3	12
High CAA (≥8)	M	68	4/4	3	15	C3	3	15	B3	VI	High	2	9
High CAA (≥8)	F	93	4/4	3	14	C3	3	14.5	B3	VI	High	2	9
High CAA (≥8)	M	83	4/4	3	15	C3	3	15	B3	VI	High	2	9
High CAA (≥8)		58			14.5	C3		14	B3	VI	High		8
High CAA (≥8)		60			12.5	C3		15	B3	VI	High		12
High CAA (≥8)		90			14	C3		14.5	B3	VI	High		9
High CAA (≥8)	F	85	3/3	3	14	C3	3	15	B3	VI	High		
Aged control	F	83	2/3	2.5	10	C2	0	1.5	B1	II	Criteria not met	2	8
Aged control	M	80	2/2	0	4.5	C1	0	5	B2	III	Criteria not met	0	0
Aged control	F	87	2/3	1.5	4.5	C2	0	4.25	B2	III	Criteria not met	0	0
Aged control	M	97	2/3	0	0	C0	0	5	B2	III	Criteria not met	0	0
Aged control	F	91	2/3	0	0	C0	0	5	B2	III	Criteria not met	0	0
Aged control	M	85	3/3	0	0	C0	0	1	B1	I	Criteria not met	0	0
Aged control	M	92	3/3	1.5	8	C2	0	4.5	B2	III	Criteria not met	3	11
Aged control	M	89	3/3	0	0	C0	0	2	B1	II	Criteria not met	0	0
Aged control	M	81	3/3	0	0	C0	0	4.5	B2	III	Criteria not met	0	0
Aged control	M	80	3/3	0	0	C0	0	4	B2	III	Criteria not met	1	6
Aged control	F	88	3/4	0	0.25	C0	0	2	B1	II	Criteria not met	0	0
Aged control	M	97	3/4	0	0	C0	0	5	B2	III	Criteria not met	0	0
Aged control	M	87	3/4	2	8.25	C2	0	2.5	B2	III	Criteria not met	0	0
Aged control	F	80		0	0	C0			B2	III	Criteria not met	0	0
Aged control	F	94		0	0	C0			B2	III	Criteria not met	0	0

**Table:** 

**Groups**	**Gender (Male)**	**Age (yrs)**	**ApoE4 allele carrier**	**Last MMSE score**	**NIA-R (High)**	**Neuritic plaque (C3)**	**NFT stage (B3)**	**TMT 10 PLEX LABELS**
Young controls (*N* = 9)	56% (5/9)	47 ± 3.3	67% (6/9)	N/A	N/A	N/A	N/A	−126, −127N, −128C
Aged controls (*N* = 15)	60% (9/15)	87.4 ± 1.5	20% (3/15)	28.1 ± 0.5	0% (0/15)	0% (0/15)	0% (0/15)	−126, −127N, −127C*, −128N
Low CAA Score (≤4) [AD] (*N* = 10)	30% (3/10)	85.5 ± 2.9	40% (4/10)	16.7 ± 2.4	50% (5/10)	60% (6/10)	50% (5/10)	−129C,−130N, −130C, −131
High CAA Score (≥8) [AD] (*N* = 10)	40% (4/10)	81 ± 4.4	60% (6/10)	14 ± 3.3	90% (9/10)	100% (10/10)	90% (9/10)	−129N, −129C,−130N, −130C, −131

*Cx, Cortex; ADNC, Alzheimer's disease neuropathological change; NIA-R, National institute of Aging Regan Diagnosis of AD*.

### Isolation of Enriched Cerebrovessel Fractions

The enriched cerebrovasculature was isolated from the inferior frontal gyrus brain tissue as previously characterized and described by our group (Alonzo et al., 1998; Ojo et al., 2021). Briefly, frozen blocks of brain tissue (500 mg) from the inferior frontal gyrus was homogenized in ice-cold Hanks Buffered salt solution (HBBS) in a glass dounce homogenizer, using 6-8 passes of a Teflon pestle tissue grinder. Forty percent dextran solution was added to the brain homogenate at an equal volume in a 15 ml falcon tube, to generate a final concentration of 20% dextran, which was subsequently centrifuged at 6,000 g for 15 mins at 4°C. Three visible layers were produced in the 15 ml falcon tube after centrifugation; the top layer consisted of a compact mass (i.e., paraenchyma fraction), the bottom layer consisted of a tissue pellet (i.e., the cerebrovasculature fraction), and this was separated by a middle layer of translucent dextran interface (i.e., non-cell associated soluble fraction). For subsequent analyses we used the bottom layer consisting of the whole cerebrovascular fraction, containing vessels of a variety of sizes (microvessels, arterioles, etc.). This fraction is highly enriched in endothelial and mural cells (i.e., pericytes and smooth muscle cells) and other perivascular cell types (e.g., astrocytes etc.).

### Subcellular Protein Extraction From Vascular Homogenates

Two hundred and fifty microliters of ice cold phosphate buffered saline (PBS) was added to each cerebrovascular pellet, followed by homogenization using a probe sonicator and subsequent centrifugation at 20,000 g for 5 mins at 4°C. Supernatant was collected in a different Eppendorf tube to obtain the PBS-fraction. Pelleted samples were re-suspended in 250 ul of ice cold PBS containing 1M sodium chloride, further sonicated and centrifuged at 20,000 g for 5 mins at 4°C. Supernatant was collected in a different Eppendorf tube and labeled as PBS-high salt fraction. The precipitant was resuspended in 250 ul of ice cold 20 mM Triethylamonium bicarbonate (TEAB) and 2% lithium dodecylsulphate anionic detergent, sonicated, and also followed by centrifugation at 20,000 g for 5 mins at 4°C. Final supernatant was transferred to a new Eppendorf tube and labeled as the membrane protein pellet fraction. PBS, PBS-high salt and membrane fractions (i.e., cytosolic, nuclear and membrane proteins) were used in the entire study to enhance the proteomic mining process.

### Trypsin Digestion

Twelve and a half microliters of 21x proteinase inhibitor cocktail was added to 250 ul of the PBS, PBS-high salt and membrane protein fractions, followed by BCA analyses to determine protein concentration. For the **PBS fraction**, 30 ug protein was added to 3x volume of acetone, and left to incubate at −20°C for 1 h. Following centrifugation at 14,000 g for 1.5 mins at room temperature, pelleted samples were brought up in 2 0ul modified reduction alkylation buffer (MRAB) consisting of 20 mM TEAB at pH 8, 1% w/v sodium deoxycholate (SDC), 1 mM tris (2-carboxyethyl) phosphine (TCEP), and 2.5 mM 2-chloroacetamide (CAM). For the **PBS-high salt fraction**, 30u g protein was added to 1 in 5 parts of 20% w/v Trichloroacetic acid (TCA) and 3x volume of acetone, and left to incubate on ice for 1 h. Following centrifugation at 14,000 g for 1.5 mins at room temperature, pelleted samples were washed with 200 ul of acetone and pelleted material brought up in 20 ul MRAB. For the **membrane protein pellet fraction**, 30 ug protein was added to 20% of 100% w/v Trichloroacetic acid (TCA), and left to incubate on ice for 1 h. Following centrifugation at 14,000 g for 1.5 mins at room temperature, pelleted samples were also washed with 200 ul of acetone and pelleted material resolubilized in 20 ul of MRAB. Validation of protein separation in all three protein fractions was conducted using sypro-red and Coomassie staining for total protein after polyacrylamide gel electrophoresis. Seven and a half microliters of all three protein fractions underwent trypsin digestion at a 1:100 enzymatic concentration. Firstly, re-suspended samples in MRAB were incubated at 37°C for 30 min; 7.5 ul of prepared activated trypsin solution (Promega, WI, USA) was added to re-suspended samples, and further incubated overnight at 37°C while shaking mildly. Digested samples were stored at −80°C prior to TMT labeling.

### Tandem Mass Tag (TMT) Labeling Strategy

We used a multiplexed isobaric labeling approach to allow for simultaneous identification and quantification of proteins from multiple biological samples. A 10-plex TMT labeling kit (ThermoScientific, NJ, USA) was used for analyses of protein samples from AD/controls and Aged/Young and an aged-matched control sample was used as a reference sample per plex for normalization of data and as a reference point for the different runs. This labeling strategy allowed for all different groups to be randomized and analyzed within the same batch. All samples and isobaric label tags were handled blind to the experimenter. Twenty microliter aliquots of each label (dissolved in 20 ul of acetonitrile solution) were dried down in the speed vacuum and re-suspended in 25 mM TEAB made up in acetonitrile solution. Re-suspended labels were subsequently added to 10 ul of dried digested protein samples, and allowed to incubate for 1 h at room temperature, after which 1 ul of formic acid solution was added to stop the reaction. Labeled samples were pooled together in entire batches and subsequently dried in the speed vacuum.

### Sodium Deoxycholate (SDC) and Tetraethylammonium Bromide (TEAB) Clean Up

To remove traces of SDC and TEAB, protein samples were re-suspended in 100 ul of 1% formic acid solution and centrifuged at 15,000 rpm for 1 min to allow separation into different phases. Supernatants were collected in new Eppendorf tubes, and 200 ul of ethyl acetate was added, and centrifuged at 15,000 rpm with the upper organic layer discarded. This process was repeated three separate times, with the final lower phase taken to dryness in the speed vacuum. The resultant dried samples were re-solubilized in 100 ul of 0.1% formic acid.

### Purification and Concentration of Peptides

Prior to ultra-high pressure liquid chromatography (UHPLC), single step desalting, concentration and purification of peptides were conducted using 0.6*u*l C18 resin Ziptips (ThermoScientific, NJ, USA). Briefly, ziptips pipette tips were used to remove contaminants by aspirating and dispensing in a solution of 0.1% formic acid made up in 50% acetonitrile (i.e., wetting buffer), and afterwards in a solution containing 0.1% formic acid (i.e., binding buffer). Ziptips were used for sample binding, by aspirating and dispensing through the samples multiple times. The resultant concentrated and purified labeled samples were aspirated in a solution of 5% methanol and 0.1% formic acid (i.e., washing buffer), followed by elution in a solvent containing 10 ul of 0.1% formic acid made up in 50% acetonitrile (wetting buffer). After desalting and concentrating peptides, final samples were dried and re-suspended in 20 ul of 0.1% formic acid and subsequently transferred into an auto-sampler vial, and analyzed by nano-Ultra-Performance Liquid Chromatography (UPLC) MS on a Q-Exactive Orbitrap instrument (ThermoScientific, NJ, USA).

### Chromatography and Mass Spectrometry (LC-MS/MS) Methods

Protein samples were analyzed using LC-MS/MS (Q-Exactive). Data dependent acquisition (DDA) settings for these MS experiments followed our previous work (Ojo et al., 2020; Pearson et al., 2020). DDA settings were as follows: full-scan MS resolution = 140 000 full width at half maximum at 200 m/z, full-scan range = 380–1250 m/z, isolation width = 1.2 m/z, higher energy C-trap dissociation relative collision energy = 29, a minimum m/z setting of 100 m/z was used for all MS^2^ spectra, MS^2^ resolution = 17 500, dynamic exclusion = 180 s, and a Top 15 high/low duty cycle was used for precursor ion selection. We used a narrow isolation window and an ultra-long gradient setting to minimize the deleterious effects on quantitative accuracy that typically result from co-isolation of isobaric precursors without resorting to MS^3^-based method.

### Data Processing and Statistical Analysis of Proteomics Data

We surveyed our amalgamated data-files and added other modifications to our search criteria if deemed necessary, using the PMi preview software. Preview results were used to choose the precursor and fragment ion mass tolerances (4-ppm, 0.02-Da, respectively) and dynamic modifications. We used the following settings to search the data using SEQUEST and BYONIC as the search algorithms, and Uniprot human database (FEB/2018). Dynamic modifications –Oxidation/+15.995 Da (M), Methyl/+14.016 Da (E), Deamidated/+0.984 Da (N, Q), static modifications of TMT10plex/+229.163 Da (N-Terminus, K), Carbamidomethyl +57.021 (C). Only unique peptides were considered for our final quantification. We used the Percolator feature of Proteome Discoverer for SEQUEST, and used the target-decoy feature, to set a false discovery rate (FDR) of 0.01 for Byonic. The peptides passing this stringent cutoff FDR rate were subsequently exported for data cleaning and statistical analysis. Master proteins only underwent quantitative analysis if they were identified in at least 50% of the total number of plexes. Log2 fold change and expression *p*-value between the two groups of interest after log transformation and parametric analyses were subsequently uploaded into ingenuity pathway analyses (IPA) where molecules and pathways, diseases and biofunctions, associated networks and upstream regulators unique to each group comparison(s) were identified. Only master proteins with a significant non-adjusted *p*-value (cut-off <0.05) were uploaded into IPA. We have deposited the mass spectrometry proteomic data into the ProteomeXchange Consortium via the PRIDE partner repository (Vizcaíno et al., 2016). Our datasets can be located with the unique identifier—PXD023340.

### Ingenuity Pathway Analysis

All datasets of significantly modulated proteins from our group comparisons were uploaded into the Ingenuity Pathway Analysis software [IPA, Ingenuity® Systems (Krämer et al., 2014)] to map them onto known networks of protein interactions in the knowledgebase. We further used the IPA knowledgebase to further determine the significantly regulated Canonical pathways/disease and biofunctions and biological significance of CAA/AD and age dependent changes in the cerebrovasculature. Our core analysis settings involved the following—Ingenuity Knowledge base as reference set, maximum number of 35 molecules per network, and a maximum number of 25 networks for analysis. Only experimentally observed knowledge was considered in our analyses. We controlled for data sources, species, and tissue type/cell lines at the time of analysis in IPA. Core analysis identified canonical pathways shown to be significantly altered in response to age and CAA/AD pathogenesis as a result of significantly regulated proteins represented in those pathways/biofunctions. Statistical significance of the relationship between uploaded dataset and the identified pathways/biofunctions was measured using two methods: 1) Ratio of the number of molecules from the data set that map to a pathway/biofunction divided by the total number of molecules in that pathway/ biofunction knowledgebase in IPA. 2) Fisher's exact test, to calculate a *p*-value determining the probability that the association between the proteins in the dataset and the pathway/biofunctions are explained by chance alone. *P*-values were considered to be significant in these studies when *P* < 0.01. Upstream regulator analysis was used to predict the upstream transcriptional master regulators in our proteomic dataset, and this was generated using the Ingenuity® Knowledge Base. An overlap P^*^value was generated based on analyses of the significant overlap between proteins/genes in our dataset and known targets modulated by the transcriptional regulator or Upstream master regulator. The activation z-score algorithm was used to make predictions. For the network analysis, a p-score [–log10 (*p*-value)] according to the fit of the set of supplied proteins and a list of biological functions stored in the Ingenuity Knowledge Base were generated. An experimentally set confidence with only the inclusion of human data and a size constraint of 35 focus molecules per network was applied as described above. We considered both direct and indirect relationships for the network analysis.

## Results

### Demographics and Clinical Background of Patient Population

In this study, we used 44 total brain cerebrovascular specimens from the inferior frontal gyrus obtained from young healthy controls (9 cases), aged non-demented controls (15 cases) and Alzheimer's disease (AD) patients (20 cases; 10 cases each with Low or High CAA score) — (Table 1).

Most of the AD cases and matched aged non-demented control cases comprised of septuagenarians and octogenarians (Table 1). On average, aged non-demented control cases (87.4 ± 1.5 yrs) were older than both AD cases with low (85.5 ± 2.9 yrs) and high (81 ± 4.4 yrs) CAA scores. While, the young control group had an average age of 45.83 ± 4.39 yrs. Each group consisted of mixed genders, and on average, there were more female in the AD cases (30-40% males) than the control cases (56-60% males)—(Table 1).

AD cases with low or high CAA scores consisted of 40% or 67% APOE4 allele carriers, respectively; while only 20% of aged non-demented control cases were APOE4 allele carriers. The young healthy control cohort consisted of 60% APOE4 allele carriers (Table 1).

**Figure 1:**
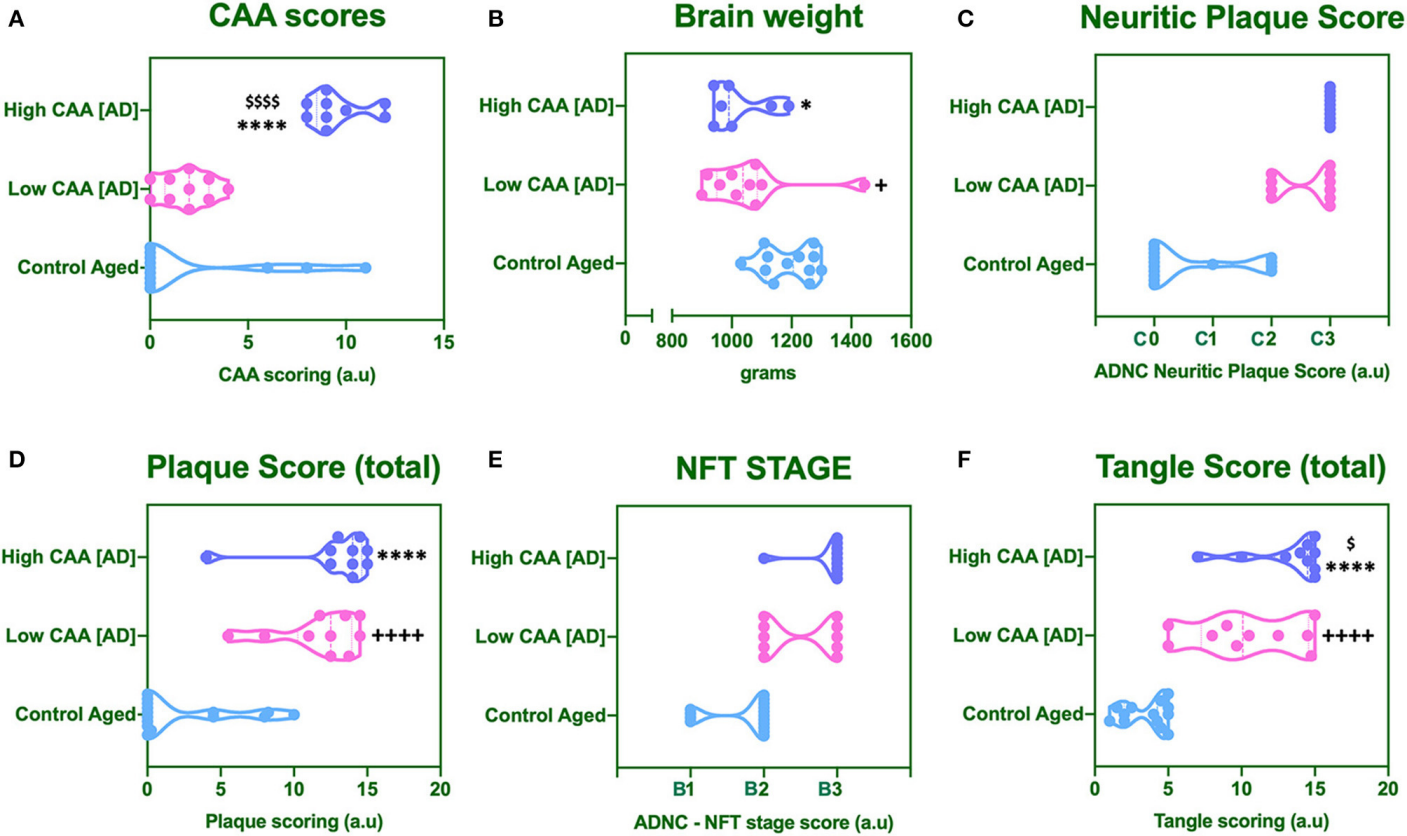
Cerebral amyloid angiopathy [CAA] **(A)**, mean brain weight in grams **(B)** and neuropathological scores for Neuritic Plaque **(C)**, Total Amyloid plaque score **(D)**, NFT staging **(E)**, and total tangle pathology score **(F)**. Data was analyzed by one way ANOVA with Holm-Sidak *post-hoc* test. *P < 0.05 and ****P < 0.0001 (for Control Aged vs. High CAA group); ^**+**^*P* < 0.05 and ^++++^*P* < 0.0001 (for Control Aged vs. Low CAA group); ^$^*P* < 0.05, and ^$$$$^*P* < 0.0001 (for Low vs. High CAA group).

No statistical difference was observed in CAA score for AD cases with low CAA staging compared to their age matched counterparts (Figure 1A). However, there was a significant reduction in brain weight (Figure 1B), and a significant increase in Amyloid Plaque and Tangle pathology in low CAA [AD] cases compared to matched controls (Figures 1C–F).

There was a significant reduction in the brain weight of high CAA compared to aged matched controls (Figure 1B). There was also a significant increase in Amyloid plaque and Tangle pathology (Figures 1C–F), accompanied by the lowest performances in the last MMSE scores (see Table 1).

Only Tangle pathology was found to be statistically significant between low vs. high CAA Alzheimer's disease cases (Figure 1F).

### Proteomic Profiles, Cell Type Specific Changes, Altered Canonical Pathways and Upstream Regulators Between Young vs. Aged Control Cerebrovascular Tissue

**Figure 2:**
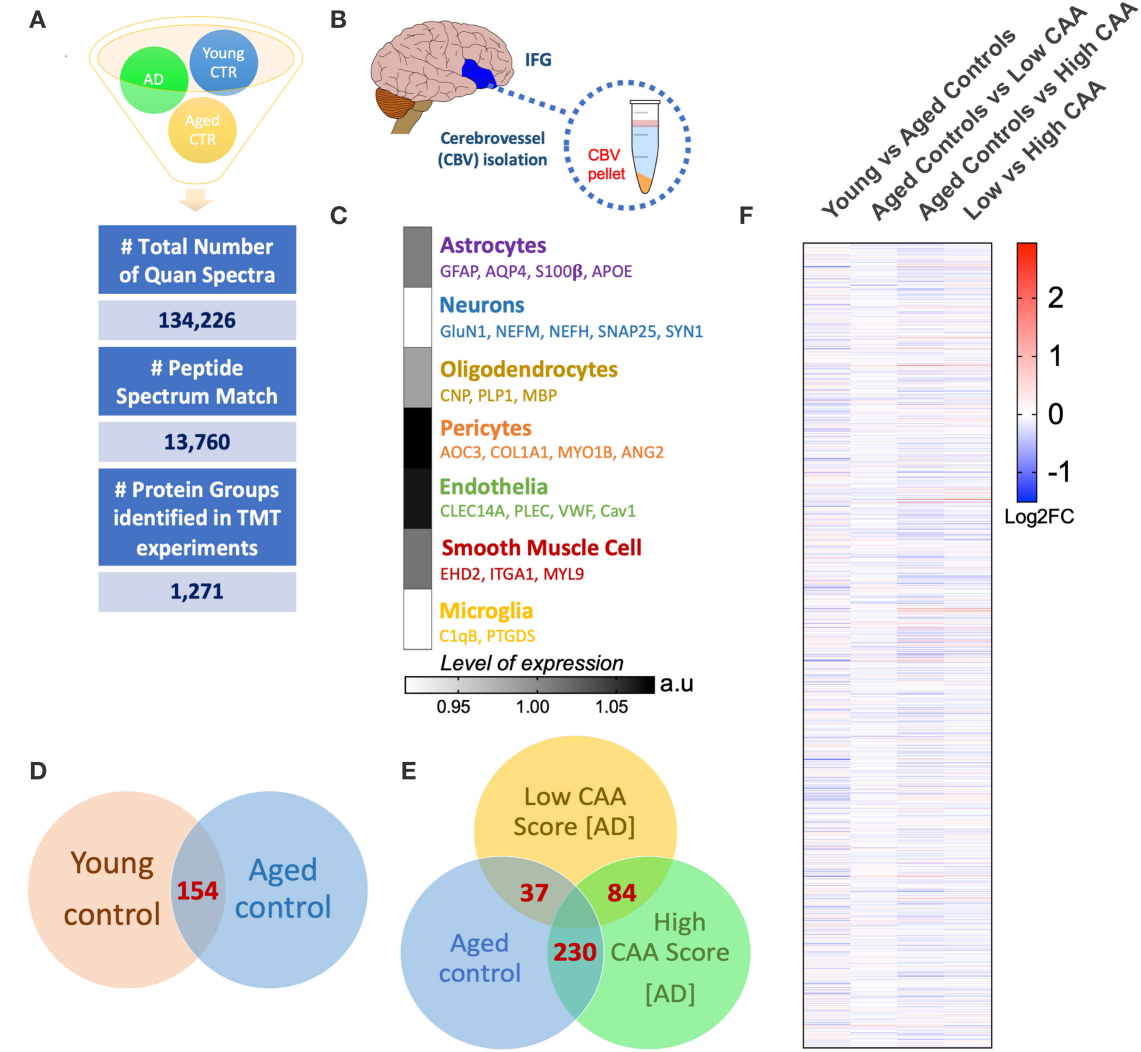
Summary of liquid chromatography/mass spectrometry (LC/MS) and proteomic analyses of isolated cerebrovascular tissue from the inferior frontal gyrus in Alzheimer's disease (AD) and young/aged matched control cases. **(A)** Shows identified total number of quantified spectra, peptide spectrum matches and non-redundant master protein groups from all plexes used for quantitative proteomic analyses of **(B)** isolated cerebrovascular tissue from the inferior frontal gyrus [IFG]. **(C)** Data shows expression levels of distinct genes associated with specific cell types identified from our proteomic analyses of the isolated cerebrovasculature. Data represent abundant ratio expressed in arbitrary units. Venn diagram in **(D,E)** shows overlapping significantly regulated proteins by *t*-test in the comparisons between young vs. aged healthy controls cases and Low CAA vs. High CAA vs. Age-matched controls, respectively. **(F)** Shows heat map of proteins identified from our proteomic analyses between young vs. aged controls, aged control vs. low CAA, aged controls vs. high CAA and low vs. high CAA groups (data represent Log2 fold change).

A Ten multiplex TMT isobaric tag approach was used to study the proteomic profiles of brain cerebrovascular tissue from the inferior frontal gyrus of AD and non-demented young and aged-matched control cases. We identified a total of 13,760 total peptide spectrum matches and 1,271 non-redundant master protein groups (Figure 2A). To determine the cell type constituents of our enriched cerebrovascular tissue we identified cell specific protein markers in our proteomic dataset using the single cell sequencing resource from the PanglaoDB omic database. We measured the relative protein expression levels of these specific markers associated with these different cell types in control cases, and observed that there was a relatively high expression of markers associated with pericytes (AOC3, COL1A1, MYO1B) and endothelia (CLEC14A, VWF, Cav1, PLEC) in our enriched cerebrovascular fractions (Figures 2B,C). In rank order, this was followed by smooth muscle cells (EHD2, ITGA1, MTL9), astrocyte (GFAP, AQP4, S100β, APOE) and oligodendrocytes (CNP, PLP1, MBP)–Figure 2C. Neurons and microglia specific markers were also observed but showed very low expression levels relative to the other cell types above (Figure 2C).

**Figure 3:**
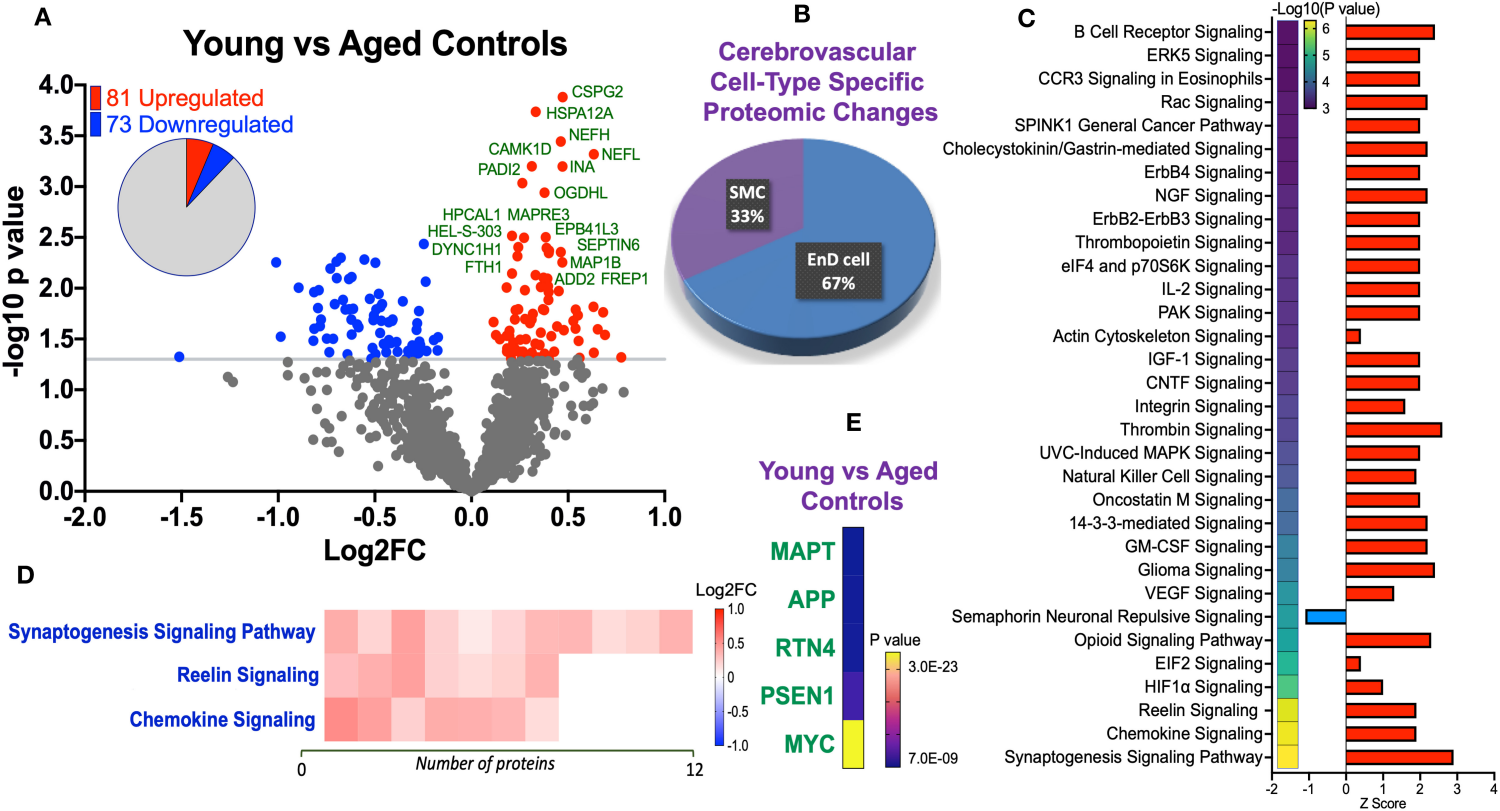
Proteomic changes, cell origin, signaling pathways, upstream regulator factors observed in the cerebrovasculature isolated from the inferior frontal gyrus of young and aged controls. **(A)** Volcano plot of differentially expressed proteins in young and aged controls (pie chart inset shows up/down-regulated proteins, significant cut off set at 1.3 and red and blue points indicated up- or down-regulated significant proteins, respectively). **(B)** Pie Chart show origin of cell types where significant proteins from the comparisons between young and aged controls are observed. Data are generated from the number of significantly regulated proteins per specific cell type (from the PanglaoDB omic database), expressed as a percentage. **(C)** Canonical pathways identified from ingenuity pathway analyses [data depict –log10 (*P*-value) and Z score generated from Fischer test of an overlap with the IPA knowledgebase; blue—downregulated and red—upregulated], and **(D)** shows heat map of the top 3 pathways and the corresponding number of significantly regulated proteins altered per pathway and their Log2 fold change expression level. **(E)** Shows Top 5 identified upstream regulators from the ingenuity pathway analyses of differentially regulated proteins in young vs. aged control cases.

Statistical analyses of the proteomic datasets first involved comparing significant changes in master proteins between young vs. aged control cases. We noted 154 significant proteins out of 1,271 that were significantly altered between young vs. aged control cases (see Venn diagram in Figures 2D,F). Volcano plot in Figure 3A depicts the correlation between Log2 fold change and negative Log10 *p*-value of the (total and significantly regulated) master proteins identified in our proteomic analyses; 81 proteins were significantly upregulated while 73 proteins were significantly downregulated (Figure 3A; see *pie chart inset*). The List of the Top 20 proteins significantly altered between young vs. aged control cases is shown in Table 2. For a complete list of all proteins, see Supplementary Table 1, corresponding protein network interaction can also be found in Supplementary Figure 1.

**Table 2:** List of Top 25 proteins significantly regulated in the inferior frontal gyrus cerebrovasculature of young and aged control cases.

**Gene name**	**Uniprot ID**	**Protein name**	**Biological function**	**Log2FC**	**–Log10 (*P*-value)**
CSPG-2	Q59FG9	Chondroitin sulfate proteoglycan 2 (Versican)	Cell adhesion	0.47	3.88
HSPA12A	A0A1B0GTF3	Heat shock 70 kDa protein 12A	Heatshock proteins (found in atherosclerotic lesions)	0.33	3.74
NEFH	P12036	Neurofilament heavy polypeptide	Axon development	0.46	3.44
NEFL	P07196	Neurofilament light polypeptide	Anterograde axonal transport	0.63	3.32
CAMK1D	Q8IU85	Calcium/calmodulin-dependent protein kinase type 1D	Inflammatory response	0.31	3.2
INA	Q16352	Alpha-internexin (Alpha-Inx) (66 kDa neurofilament protein)	Cell differentiation	0.47	3.2
PADI2	Q9Y2J8	Protein-arginine deiminase type-2	Cellular response to leukemia inhibitory factor	0.26	3.03
OGDHL	Q9ULD0	2-oxoglutarate dehydrogenase-like, mitochondrial	Glycolytic process	0.38	2.94
HPCAL1	P37235	Hippocalcin-like protein 1	Calcium binding protein	0.21	2.52
MAPRE3	B2R5W6	Microtubule-associated protein, RP/EB family, member 3	Cell cycle	0.38	2.5
FTH1	Q6NS36	Ferritin (Fragment)	Cellular iron ion homeostasis	0.27	2.5
HEL-S-303	V9HW12	Epididymis secretory protein Li 303	Activation of MAPK activity	−0.25	2.44
EPB41L3	Q9Y2J2	Band 4.1-like protein 3	Actomyosin structure organization	0.24	2.4
MAP1B	A0A024RAM4	Microtubule-associated protein 1B	Microtubule cytoskeleton organization	0.39	2.4
ADD2	P35612	Beta-adducin (Erythrocyte adducin subunit beta)	Actin cytoskeleton organization	0.4	2.37
FREP1	Q4L233	Brain-specific protein p25 alpha (Fibroblast growth factor-2 repression protein-1)	Microtubule bundle formation	0.46	2.36
SEPTIN6	Q8NFH9	MLL/SEPTIN6 fusion protein	Histone methyltransferase activity	0.4	2.35
DYNC1H1	Q14204	Cytoplasmic dynein 1 heavy chain 1	Antigen processing and presentation of exogenous peptide antigen via MHC class II	0.24	2.32
ERP29	P30040	Endoplasmic reticulum resident protein 29	Activation of MAPK activity	−0.68	2.3
KHDRBS1	Q07666	KH domain-containing, RNA-binding, signal transduction-associated protein 1	G1/S transition of mitotic cell cycle	−0.55	2.28
PRPF40A	O75400	Pre-mRNA-processing factor 40 homolog A (Fas ligand-associated factor 1)	Cell cycle	−0.7	2.26
PCSK1N	Q9UHG2	ProSAAS (Proprotein convertase subtilisin/kexin type 1 inhibitor)	Neuropeptide signaling pathway	−1.01	2.25
CK	B4DP56	Creatine kinase	Phosphocreatine biosynthetic process	0.47	2.25
HNRNPA3	P51991	Heterogeneous nuclear ribonucleoprotein A3	mRNA splicing, via spliceosome	−0.5	2.25
NSAP1	B2R8Z8	Synaptotagmin binding, cytoplasmic RNA interacting protein	mRNA stability	−0.73	2.19

*Data are expressed as the negative Log10 of the p-value (significance cut off set at >1.3 or P < 0.05), and the Log 2 fold change between young vs. aged control cases*.

**Figure 4:**
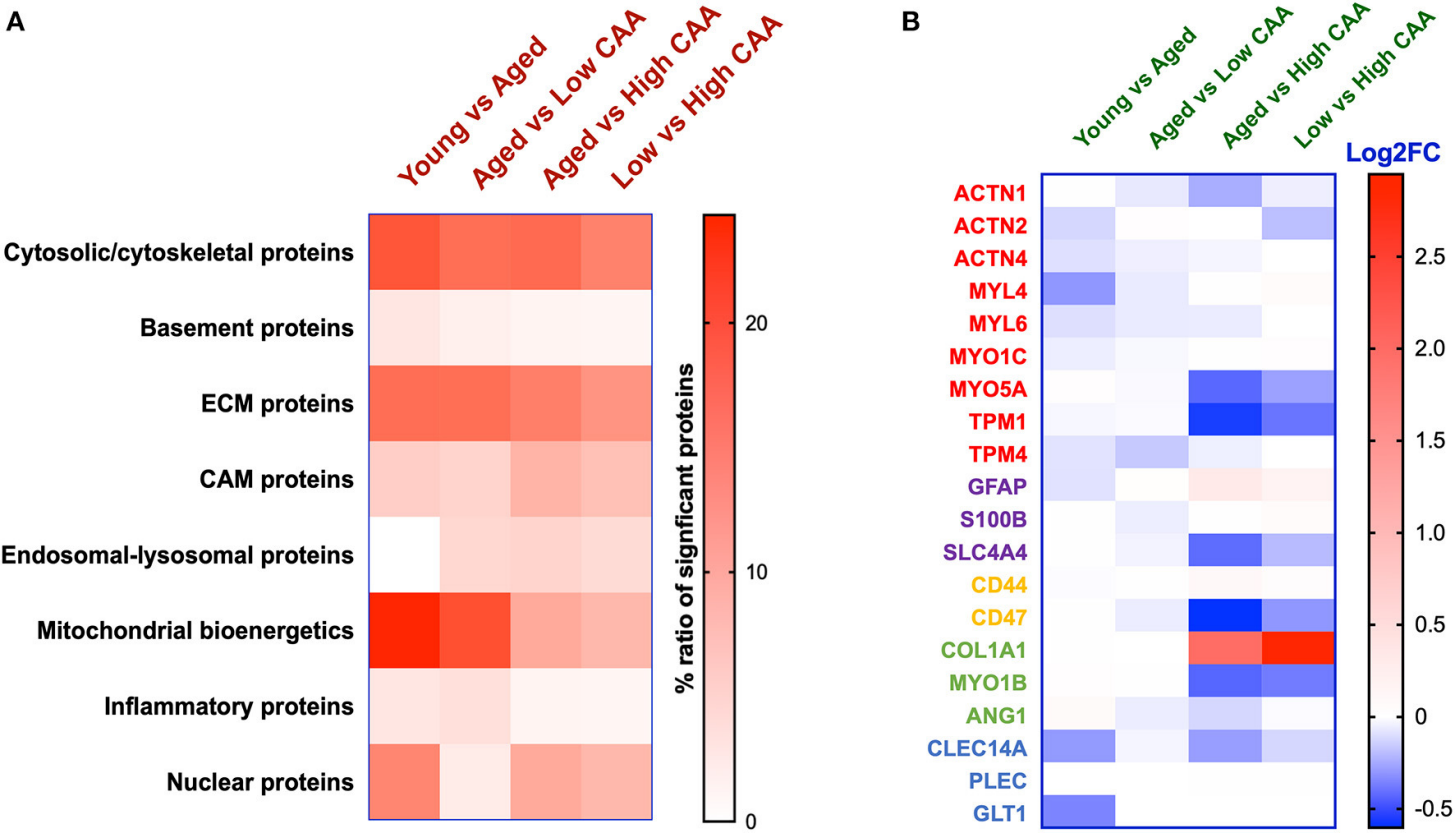
Ratio of significantly regulated proteins per subcellular localization or biological function and Cell specific proteins expression levels identified in cerebrovasculature of young and aged controls, and AD cases staged by low vs. high CAA score. **(A)** Data shows percentage of significantly altered proteins associated with a biological function or subcellular localization. ECM—Extracellular matrix protein, CAM—cellular adhesion molecule. **(B)** Data shows cell specific proteins expression levels. Proteins in red represents smooth muscle cell markers, purple (astrocytes), yellow (microglia), pericytes (green), blue (endothelial cells). Data shows Log2 fold change (*note: not all cell specific proteins depicted passed the set cut-off value of P* < *0.05*).

The majority of these proteomic changes were attributed to cytoskeletal, cytosolic, nuclear and extracellular cell membrane proteins, including proteins associated with mitochondrial bioenergetics (Figure 4A).

From the list of 154 proteins significantly regulated, we identified the number of proteins that were known to specifically originate from a particular cerebrovascular cell type, and expressed this as a percentage, to capture the specific contribution of each cerebrovascular cell type in our proteomic findings. We observed that most of the significant changes (*P* < 0.05) in our proteomic datasets between young vs. aged control cases were associated with endothelial cell specific markers (67%) and smooth muscle cell markers (33%) (Figures 3B, 4B).

Ingenuity pathway analyses (IPA) identified 32 pathways significantly impacted between young vs. aged control cases (Figure 3C). The top 3 pathways include downregulation of synaptogenesis signaling pathways, Reelin signaling and Chemokine signaling. Heat map in Figure 3D, shows the number of proteins associated with these Top three pathways, and their directionality of change.

We interrogated the Top 5 Upstream Regulators mediating the changes observed in significantly regulated proteins identified from our analyses (Figure 3E). IPA analyses identified a significant overlap in our dataset and known targets regulated by these five Upstream regulators, namely (in rank order of significance)–MAPT (microtubule associated protein tau), APP (amyloid precursor protein), RTN4 (Reticulon 4 or Nogo), PSEN1 (Presenilin 1), and MYC (Proto-Oncogene, BHLH Transcription Factor).

### Proteomic Profiles, Cell Type Specific Changes, Altered Canonical Pathways, and Upstream Regulators Between AD (Low CAA Score) vs. Aged-Matched Control Cerebrovascular Tissue

**Figure 5:**
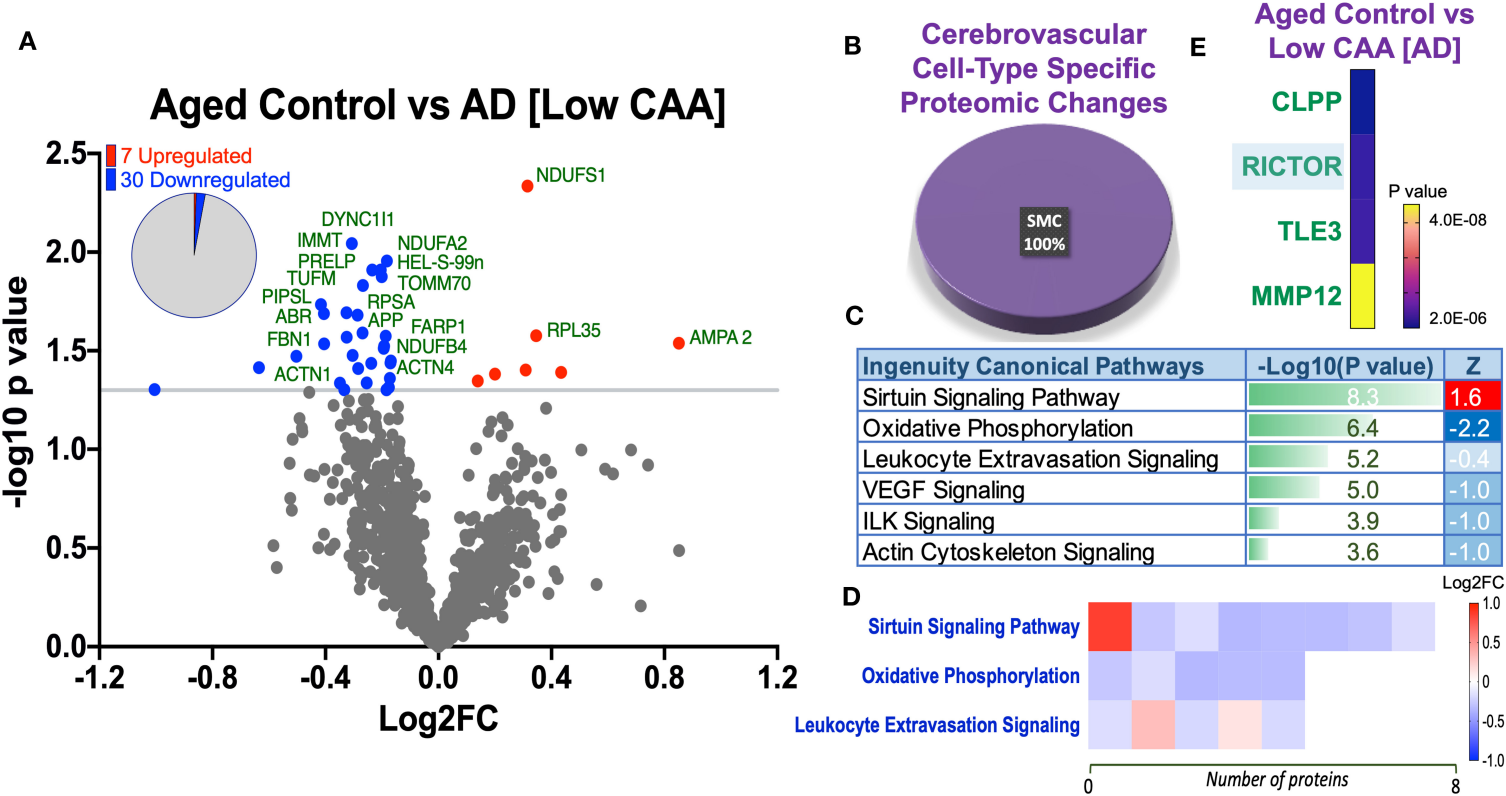
Proteomic changes, cell origin, signaling pathways, upstream regulator factors observed from the cerebrovasculature isolated from the inferior frontal gyrus of low CAA vs. aged-matched control cases. **(A)** Volcano plot of differentially expressed proteins in low CAA vs. aged-matched control cases (pie chart inset shows up/down-regulated proteins, significant cut off set at 1.3 and red and blue points indicated up- or down-regulated significant proteins, respectively). **(B)** Pie Chart show origin of cell types where significant proteins from the comparisons between low CAA vs. aged-matched control cases are observed. Data are generated from the number of significantly regulated proteins per specific cell type (from the PanglaoDB omic database), expressed as a percentage. **(C)** Canonical pathways identified from ingenuity pathway analyses [data depict –log10 [*P*-value] and Z score generated from Fischer test of an overlap with the IPA knowledgebase; blue—downregulated and red—upregulated], and **(D)** shows heat map of the top 3 pathways and the corresponding number of significantly regulated proteins altered per pathway and their Log2 fold change expression level. **(E)** Shows Top 4 identified upstream regulators from the ingenuity pathway analyses of differentially regulated proteins in low CAA vs. aged-matched control cases (*light blue highlighted text indicates that the upstream regulator is predicted to be activated*).

Despite the profound clinicopathological differences observed between AD cases with low CAA compared to their age-matched counterparts, at the vascular level, we only observed 37 significant proteins out of 1,271 that were significantly altered (see Venn diagram in Figures 2E,F). Volcano plot in Figure 5A depicts the correlation between Log2 fold change and negative Log10 *p*-value of the (total and significantly regulated) master proteins identified in our proteomic analyses; seven proteins were significantly upregulated while 30 proteins were significantly downregulated (Figure 5A; see *pie chart inset*). The List of the Top 20 proteins significantly altered between Low CAA [AD] vs. age-matched control is shown in Table 3. For a complete list of all proteins, see Supplementary Table 2, corresponding protein network interaction can also be found in Supplementary Figure 2.

**Table 3:** List of Top 25 proteins significantly regulated in the inferior frontal gyrus cerebrovasculature of low CAA vs. aged-matched control cases.

**Gene name**	**Uniprot ID**	**Protein name**	**Biological function**	**Log2FC**	**–Log10 (P value)**
ACTN2	P35609	Alpha-actinin-2 (Alpha-actinin skeletal muscle isoform 2)	Actin filament uncapping	0.32	2.33
TPM4	P67936	Tropomyosin alpha-4 chain (TM30p1) (Tropomyosin-4)	Actin filament organization	−0.31	2.04
HPCAL1	P37235	Hippocalcin-like protein 1 (Calcium-binding protein BDR-1)	Calcium binding protein	−0.18	1.96
TUFM	P49411	Elongation factor Tu, mitochondrial	Mitochondrial translational elongation	−0.2	1.91
RPSA	A0A0C4DG17	(37 kDa laminin receptor precursor) (37LRP)	Ribosomal small subunit assembly	−0.23	1.91
ACTN1	P12814	Alpha-actinin-1 cytoskeletal isoform	Actin crosslink formation	−0.2	1.88
DECR1	A0A024R9D7	2,4-dienoyl CoA reductase 1, mitochondrial, isoform	Positive regulation of cold-induced thermogenesis	−0.27	1.83
MCH-2V	Q53G34	Mitochondrial carrier homolog 2 variant	Mitochondrial metabolic pathways	−0.42	1.73
PRELP	P51888	Proline-arginine-rich end leucine-rich repeat protein	Cell aging	−0.33	1.69
PIPSL	A2A3N6	Putative PIP5K1A and PSMD4-like protein	Phosphatidylinositol phosphate kinase activity	−0.41	1.69
DYNC1I1	O14576	Cytoplasmic dynein 1 intermediate chain 1	Antigen processing and presentation of exogenous peptide antigen via MHC class II	−0.29	1.68
HDGFL3	Q9Y3E1	Hepatoma-derived growth factor-related protein 3	Microtubule polymerization	−0.27	1.59
FBN1	P35555	Fibrillin-1	Activation of protein kinase A activity	0.35	1.58
PHB	A8K401	Prohibitin	Activation of phospholipase C activity	−0.19	1.57
NDUFB4	O95168	NADH dehydrogenase [ubiquinone] 1 beta subcomplex subunit 4	Mitochondrial electron transport, NADH to ubiquinone	−0.33	1.57
APP	A0A218KGR2	Alpha-secretase C-terminal fragment (Amyloid-beta A4 protein)	Endocytosis	0.85	1.54
	A8K4W2	ATP synthase F(0) complex subunit B1, mitochondrial	ATP synthesis coupled proton transport	−0.4	1.53
MYL6	B7Z6Z4	Myosin light polypeptide 6	ATPase cellular motor protein.	−0.19	1.52
IMMT	Q16891	MICOS complex subunit MIC60 (Cell proliferation-inducing gene)	Cristae formation	−0.19	1.51
TOMM70	O94826	Mitochondrial import receptor subunit TOM70	Macroautophagy	−0.3	1.48
HEL-S-99n	V9HW25	Calreticulin	Binds misfolded proteins preventing export from the ER	−0.5	1.47
ABR	A0A1C7CYZ0	Active breakpoint cluster region-related protein	Intracellular signal transduction	−0.17	1.45
ACTN4	O43707	Alpha-actinin-4	Actin filament bundle assembly	−0.17	1.44
FARP1	C9JME2	FERM, ARHGEF and pleckstrin domain-containing protein 1	Dendrite morphogenesis	−0.24	1.44
TPR	P12270	Nucleoprotein (Translocated promoter region protein)	mRNA transport	−0.64	1.41

*Data are expressed as the negative Log10 of the p-value (significance cut off set at >1.3 or P < 0.05), and the Log 2 fold change between low CAA vs. aged-matched control cases*.

The majority of these proteomic changes were also attributed to cytoskeletal, cytosolic and extracellular cell membrane proteins, including proteins associated with mitochondrial bioenergetics (Figure 4A).

From the list of 37 proteins significantly regulated between AD (low CAA score) vs. aged-matched controls, we observed that most of the significant changes (*P* < 0.05) were associated primarily with smooth muscle cell specific markers (Figures 4B, 5B).

Ingenuity pathway analyses (IPA) identified 6 pathways significantly impacted between AD (low CAA score) vs. aged-matched controls (Figure 5C). The top 3 pathways include alteration in sirtuin signaling, oxidative phosphorylation and leukocyte extravasation signaling. Heat map in Figure 5D, shows the number of proteins associated with these Top three pathways, and their corresponding fold-change.

IPA analyses identified CLPP (Caseinolytic Mitochondrial Matrix Peptidase Proteolytic Subunit), RICTOR (rapamycin-insensitive companion of mammalian target of rapamycin), TLE3 (Transducin-like enhancer protein 3), and MMP12 (matrix metalloproteinase protein 12) as the top four Upstream Regulators driving the proteomic changes between AD (low CAA score) vs. aged-matched controls (Figure 5E).

### Proteomic Profiles, Cell Type Specific Changes, Altered Canonical Pathways, and Upstream Regulators Between AD (High CAA Score) vs. Aged-Matched Control Cerebrovascular Tissue

**Figure 6:**
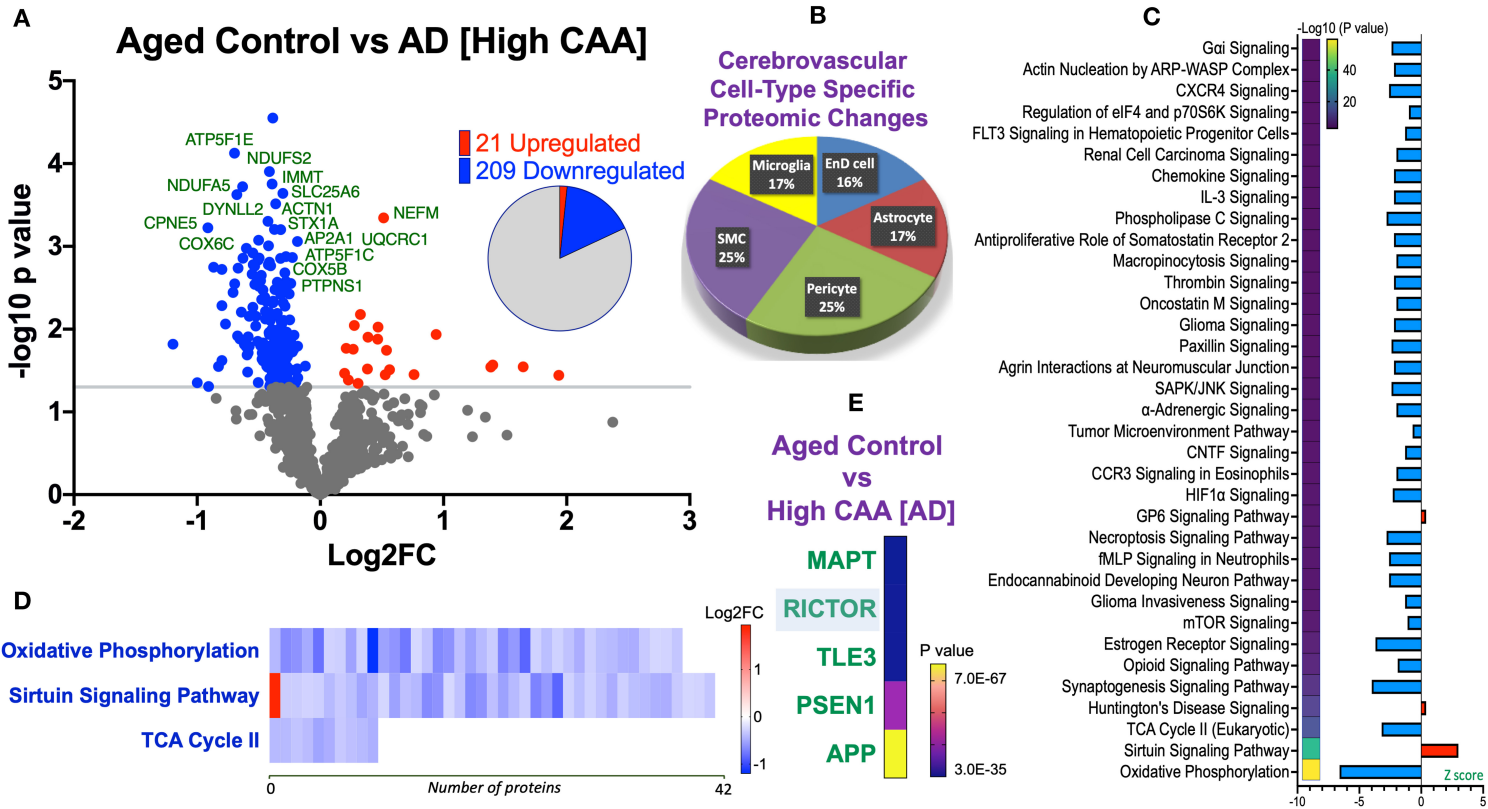
Proteomic changes, cell origin, signaling pathways, upstream regulator factors observed from the cerebrovasculature isolated from the inferior frontal gyrus of high CAA vs. aged-matched control cases. **(A)** Volcano plot of differentially expressed proteins in high CAA vs. aged-matched control cases (pie chart inset shows up/down-regulated proteins, significant cut off set at 1.3 and red and blue points indicated up- or down-regulated significant proteins, respectively). **(B)** Pie Chart show origin of cell types where significant proteins from the comparisons between high CAA vs. aged-matched control cases are observed. Data are generated from the number of significantly regulated proteins per specific cell type (from the PanglaoDB omic database), expressed as a percentage. **(C)** Canonical pathways identified from ingenuity pathway analyses [data depict –log10 (*P*-value) and Z score generated from Fischer test of an overlap with the IPA knowledgebase; blue—downregulated and red—upregulated], and **(D)** shows heat map of the top 3 pathways and the corresponding number of significantly regulated proteins altered per pathway and their Log2 fold change expression level. **(E)** Shows Top 5 identified upstream regulators from the ingenuity pathway analyses of differentially regulated proteins in high CAA vs. aged-matched control cases (*light blue highlighted text indicates that the upstream regulator is predicted to be activated*).

We noted 230 significant proteins out of 1,271 were significantly altered between AD (high CAA score) vs. aged-matched control (see Venn diagram in Figures 2E,F). Volcano plot in Figure 6A depicts the correlation between Log2 fold change and negative Log10 *p*-value of the (total and significantly regulated) master proteins identified in our proteomic analyses; 21 proteins were significantly upregulated while 209 proteins were significantly downregulated (Figure 6A; see *pie chart inset*). The List of the Top 20 proteins significantly altered between high CAA [AD] vs. age-matched control is shown in Table 4. For a complete list of all proteins, see Supplementary Table 3, corresponding protein network interaction can also be found in Supplementary Figure 3.

**Table 4:** List of Top 25 proteins significantly regulated in the inferior frontal gyrus cerebrovasculature of high CAA vs. aged-matched control cases.

**Gene name**	**Uniprot ID**	**Protein name**	**Biological function**	**Log2FC**	**–Log10 (P value)**
NDUFA9	Q16795	NADH dehydrogenase [ubiquinone] 1 alpha subcomplex subunit 9, mitochondrial	Circadian rhythm	−0.38	4.55
DECR1	A0A024R9D7	2,4-dienoyl CoA reductase 1, mitochondrial, isoform	Beta-oxidation enzyme	−0.7	4.13
NDUFB3	O43676	NADH dehydrogenase [ubiquinone] 1 beta subcomplex subunit 3	Mitochondrial electron transport, NADH to ubiquinone	−0.41	3.9
NDUFS2	O75306	NADH dehydrogenase [ubiquinone] iron-sulfur protein 2, mitochondrial	Mitochondrial ATP synthesis coupled electron transport	−0.39	3.75
ATP5F1E	P56381	ATP synthase subunit epsilon, mitochondrial	ATP biosynthetic process	−0.63	3.72
IMMT	Q16891	MICOS complex subunit MIC60 (Cell proliferation-inducing gene 4/52 protein)	Roles in the maintenance of crista junctions	−0.3	3.64
NDUFA5	A0A024R745	(NADH dehydrogenase [ubiquinone] 1 alpha subcomplex subunit 5)	Respiratory electron transport chain	−0.68	3.63
	Q59EI9	ADP,ATP carrier protein, liver isoform T2 variant	ATP transmembrane transporter activity	−0.36	3.51
NEFM	A5YM63	160 kDa neurofilament protein (Neurofilament 3)	Neurofilament bundle assembly	0.51	3.34
DYNLL2	Q96FJ2	Dynein light chain 2, cytoplasmic	Antigen processing and presentation of exogenous peptide antigen via MHC class II	−0.42	3.3
CPNE5	Q9HCH3	Copine-5 (Copine V)	Cellular response to calcium ion	−0.91	3.22
ACTN1	P12814	Alpha-actinin-1 cytoskeletal isoform	Anchors actin to a variety of intracellular structures	−0.37	3.2
UQCRC1	P31930	Cytochrome b-c1 complex subunit 1, mitochondrial	Aerobic respiration	−0.32	3.2
STX1A	Q75ME0	STX1A protein Syntaxin 1A (Brain)	Calcium-ion regulated exocytosis	−0.5	3.08
AP2A1	O95782	AP-2 complex subunit alpha−1 (100 kDa coated vesicle protein A)	Antigen processing and presentation of exogenous peptide antigen via MHC class II	−0.19	3.06
ATP5F1C	P36542	ATP synthase subunit gamma, mitochondrial	ATP biosynthetic process	−0.42	3.01
PTPNS1	D3DVW9	Protein tyrosine phosphatase, non-receptor type substrate 1	Negative regulator of the insulin signaling pathway	−0.6	2.98
COX6C	P09669	Cytochrome c oxidase subunit 6C	Generation of precursor metabolites and energy	−0.55	2.92
COX5B	P10606	Cytochrome c oxidase subunit 5B, mitochondrial	Mitochondrial ATP synthesis coupled proton transport	−0.28	2.88
PHB	A8K401	Prohibitin	Chaperone for respiration chain proteins and trancriptional regulation	−0.23	2.87
SLC4A4	A5JJ20	Anion exchange protein	Transport of anions across cellular barriers	−0.5	2.86
PRP1	B4DJ38	Pentatricopeptide repeat protein 1	Negative regulation of leucine tRNA, mitochondria-encoded proteins and COX activity	−0.51	2.86
NDUFA8	P51970	NADH dehydrogenase [ubiquinone] 1 alpha subcomplex subunit 8	Mitochondrial electron transport, NADH to ubiquinone	−0.63	2.86
PKM2	A0A024R5Z9	Pyruvate kinase	Glucose metabolic process	−0.32	2.85
ATP5MG	O75964	ATP synthase subunit g, mitochondrial (ATPase subunit g)	Catalyzes ATP synthesis during oxidative phosphorylation	−0.5	2.85

*Data are expressed as the negative Log10 of the p-value (significance cut off set at >1.3 or P < 0.05), and the Log 2 fold change between high CAA vs. aged-matched control cases*.

The majority of these proteomic changes were also attributed to cytoskeletal, cytosolic and extracellular cell membrane proteins, including also modest involvement of proteins associated with cell adhesion molecules and mitochondrial bioenergetics, and nuclear proteins (Figure 4A).

From the list of 230 proteins significantly regulated between AD (high CAA score) vs. aged-matched controls, we observed that most of the significant changes (*P* < 0.05) were associated with pericytes and smooth muscle cells (both 25%), perivascular astrocytes and microglia/macrophage (both 17%) and endothelial cell specific markers (16%) (Figures 4B, 6B).

Ingenuity pathway analyses (IPA) identified 35 pathways significantly impacted between AD (high CAA score) vs. aged-matched controls (Figure 6C). The top 3 pathways include alterations in oxidative phosphorylation, Sirtuin signaling and TCA cycle II. Heat map in Figure 6D, shows the number of proteins associated with these top 3 pathways, and their corresponding fold-change.

IPA analyses identified MAPT (microtubule associated protein tau), RICTOR (rapamycin-insensitive companion of mammalian target of rapamycin), TLE3 (Transducin-like enhancer protein 3), PSEN1 (Presenilin 1) and APP (amyloid precursor protein) as the top five Upstream Regulators driving the proteomic changes between AD (high CAA score) vs. aged-matched controls (Figure 6E).

### Proteomic Profiles, Cell Type Specific Changes, Altered Canonical Pathways, and Upstream Regulators Between Low vs. High CAA Alzheimer's Disease Cerebrovascular Tissue

**Figure 7:**
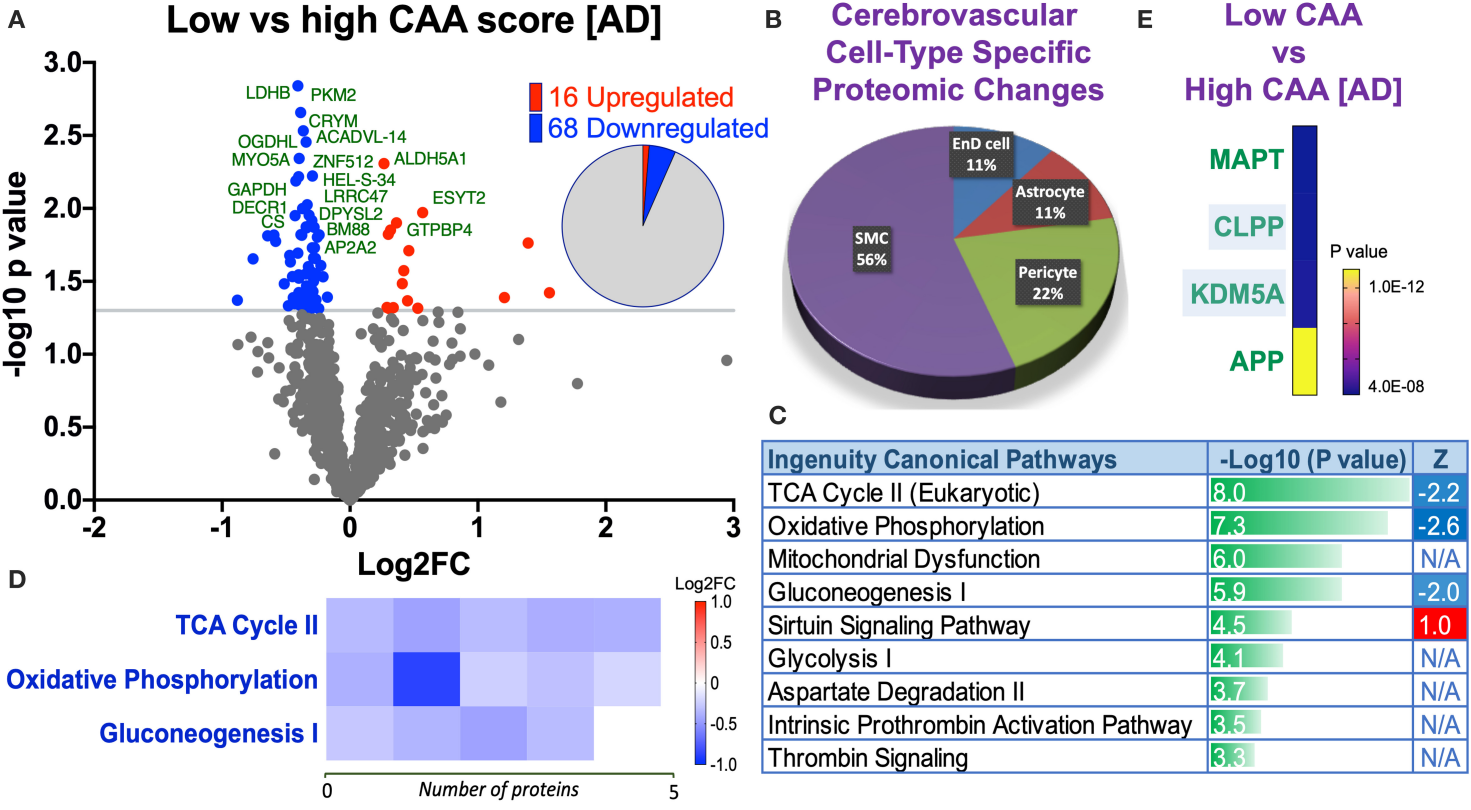
Proteomic changes, cell origin, signaling pathways, upstream regulator factors observed from the cerebrovasculature isolated from the inferior frontal gyrus of low and high CAA [AD] cases. **(A)** Volcano plot of differentially expressed proteins in low vs. high CAA [AD] cases (pie chart inset shows up/down-regulated proteins, significant cut off set at 1.3 and red and blue points indicated up- or down-regulated significant proteins, respectively). **(B)** Pie Chart show origin of cell types where significant proteins from the comparisons between low and high CAA [AD] cases are observed. Data are generated from the number of significantly regulated proteins per specific cell type (from the PanglaoDB omic database), expressed as a percentage. **(C)** Canonical pathways identified from ingenuity pathway analyses (data depict –log10 [*P*-value] and Z score generated from Fischer test of an overlap with the IPA knowledgebase; blue—downregulated and red—upregulated), and **(D)** shows heat map of the top 3 pathways and the corresponding number of significantly regulated proteins altered per pathway and their Log2 fold change expression level. **(E)** Shows Top 4 identified upstream regulators from the ingenuity pathway analyses of differentially regulated proteins in low and high CAA [AD] cases (*light blue highlighted text indicates that the upstream regulator is predicted to be activated*).

Eighty four significant proteins out of 1,271 were significantly altered between low vs. high CAA Alzheimer's disease cerebrovascular tissue (see Venn diagram in Figures 2E,F). Volcano plot in Figure 7A depicts the correlation between Log2 fold change and negative Log10 *p*-value of the (total and significantly regulated) master proteins identified in our proteomic analyses; 16 proteins were significantly upregulated while 68 proteins were significantly downregulated (Figure 7A; see *pie chart inset*). The List of the Top 20 proteins significantly altered between low vs. high CAA Alzheimer's disease cerebrovascular tissue is shown in Table 5. For a complete list of all proteins, see Supplementary Table 4, corresponding protein network interaction can also be found in Supplementary Figure 4.

**Table 5:** List of Top 25 proteins significantly regulated in the inferior frontal gyrus cerebrovasculature of low vs. high CAA [AD] cases.

**Gene name**	**Uniprot ID**	**Protein name**	**Biological function**	**Log2FC**	**–Log10 (P value)**
LDHB	Q5U077	L-lactate dehydrogenase	Carbohydrate metabolic process	−0.41	2.84
PKM2	A0A024R5Z9	Pyruvate kinase	Glycolysis	−0.39	2.66
CRYM	Q14894	Ketimine reductase mu-crystallin	Lysine catabolic process	−0.37	2.53
VLC-ACDm	B4DEA8	Very-long-chain specific acyl-CoAdehydrogenase, mitochondrial	Fatty acid and amino acid catabolism	−0.34	2.45
OGDHL	Q9ULD0	2-oxoglutarate dehydrogenase-like, mitochondrial	Degrades glucose and glutamate	−0.4	2.34
ALDH5A1	V9HWE0	Succinate-semialdehyde dehydrogenase	Negative regulation of coagulation	0.27	2.31
ZNF512	Q96ME7	Zinc finger protein 512	DNA and metal ion binding	−0.29	2.22
MYO5A	F8W6H6	Unconventional myosin-Va	Transport of vesicles to the plasma membrane	−0.4	2.22
HEL-S-34	D9IAI1	Phosphatidylethanolamine binding protein 1	Serine protease inhibitor which inhibits thrombin	−0.42	2.19
ACTN2	P35609	Alpha-actinin-2 (Alpha-actinin skeletal muscle isoform 2)	Anchor actin to a variety of intracellular structures	−0.33	2.02
GAPDH	P04406	Glyceraldehyde-3-phosphate dehydrogenase (GAPDH)	Canonical glycolysis	−0.37	2
ESYT2	A0A087WXU3	Extended synaptotagmin-2	Lipid transport	0.57	1.97
LRRC47	Q8N1G4	Leucine-rich repeat-containing protein 47	RNA binding	−0.32	1.95
DECR1	A0A024R9D7	2,4-dienoyl CoA reductase 1, mitochondrial, isoform	Beta-oxidation enzyme	−0.43	1.95
DPYSL2	A0A1C7CYX9	Dihydropyrimidinase-related protein 2	Cytoskeleton organization	−0.3	1.92
GTPBP4	D2CFK9	Nucleolar GTP-binding protein 1	Maturation of LSU-rRNA from tricistronic rRNA transcript	0.36	1.9
BM88	B2R7I3	BM88 antigen (BM88), mRNA	Neuron differentiation	−0.3	1.89
AP2A2	O94973	Adaptor protein complex AP-2 subunit alpha-2	Antigen processing and presentation of exogenous peptide antigen via MHC class II	−0.29	1.88
CS	O75390	Citrate synthase, mitochondrial	Carbohydrate metabolic process	−0.34	1.87
ALDOA	J3KPS3	Fructose-bisphosphate aldolase	Glycolytic process [GO:0006096]	−0.28	1.87
MLF2	Q15773	Myeloid leukemia factor 2	Regulation of transcription, DNA-templated	−0.34	1.87
PROSC	D3DSW3	Pyridoxal phosphate homeostasis protein	Intracellular homeostatic regulation of vitamin B6	0.32	1.85
MYO1C	O00159	Unconventional myosin-Ic (Myosin I beta)	Actin filament organization	0.3	1.82
CKMT1A/1B	P12532	Creatine kinase U-type, mitochondrial	Creatine metabolic process	−0.38	1.82
NDUFA9	Q16795	NADH dehydrogenase [ubiquinone] 1 alpha subcomplex subunit 9	Accessory subunit of the mitochondrial membrane respiratory chain	−0.24	1.82

*Data are expressed as the negative Log10 of the p-value (significance cut off set at >1.3 or P < 0.05), and the Log 2 fold change between low vs. high CAA [AD] cases*.

The majority of these proteomic changes were also attributed to cytoskeletal, cytosolic and extracellular cell membrane proteins, including also involvement of proteins associated with cell adhesion molecules and mitochondrial bioenergetics, and nuclear proteins (Figure 4A).

From the list of 84 proteins significantly regulated between low vs. high CAA Alzheimer's disease cerebrovascular tissue, we observed that most differentially significant changes (*P* < 0.05) were associated with smooth muscle cells (56%), followed by pericytes (22%), and astrocytes/endothelial cell specific markers (each 11%) (Figures 4B, 7B).

Ingenuity pathway analyses (IPA) identified 9 pathways significantly between low vs. high CAA Alzheimer's disease cerebrovascular tissue (Figure 7C). The top 3 pathways include alterations in TCA cycle II oxidative phosphorylation and mitochondrial dysfunction/gluconeogenesis. Heat map in Figure 7D, shows the number of proteins associated with these top 3 pathways, and their corresponding fold-change.

IPA analyses identified MAPT (microtubule associated protein tau), CLPP (Caseinolytic Mitochondrial Matrix Peptidase Proteolytic Subunit), KDM5A (Lysine-specific demethylase 5A) and APP (amyloid precursor protein) as the top four Upstream Regulators driving the proteomic changes between low vs. high CAA Alzheimer's disease cerebrovascular tissue (Figure 7E).

## Discussion

We employed our state-of-the-art proteomic platform to conduct a detailed unbiased characterization of changes in protein expression levels, and molecular pathways significantly altered from cerebrovascular tissue isolated from the inferior frontal gyrus of young and aged controls and AD brains staged by CAA severity. Endothelial and mural cells, supported by other perivascular cell types (e.g., perivascular astrocytes), predominantly comprise the cerebrovascular fraction isolated from these autopsy specimens. Our analyses revealed unique proteomic changes and molecular pathways driven by age and CAA severity in the sequelae of AD pathogenesis.

Specifically, we observed 150 significantly regulated proteins in young vs. aged control cerebrovessels. With respect to the cell specific markers identified from this group comparison, we noted that significant changes were mainly associated with smooth muscle and endothelial cells. Chemokine and reelin signaling, synaptogenesis signaling, hypoxia inducible factor 2α (HIF2α) and Eukaryotic initiation factor 2 (EIF2) signaling pathways were the top 5 pathways significantly upregulated with age, thus indicating that inflammation, alteration in cell to cell communication, protein-synthesis and oxidative stress may be prominent features of cerebrovessel aging. It is worthy of note that synaptogenesis was identified as one of the top signaling pathways altered. The reason behind this change remains unknown. Neuronal end-feet processes are known to connect with endothelial cells to regulate neurovascular coupling, however, our initial analyses showed very low expression levels of neuronal specific markers in the cerebrovascular fractions compared to endothelial and mural cell specific markers. An alternative explanation is that other enriched cell types such as astrocytes (Farhy-Tselnicker and Allen, 2018) or endothelial cells (Wu et al., 2019) could be the mediators of these synaptogenesis signaling pathways.

We also report unique proteomic changes in cerebrovessels of AD patients compared to age-matched control counterparts. Firstly, we stratified AD cases into two distinct groups based on low (<4) and high CAA (>8) scores. Generally, AD patients with high CAA staging had the worst MMSE results, the smallest brain weight, and most severe amyloid plaque and tau pathology compared to AD cases with low CAA staging and age-matched controls. From our analyses, we observed a significantly greater proteomic response in cerebrovessels between AD cases with high CAA staging vs. age-matched controls (230 significantly regulated protein), compared to AD cases with low CAA staging vs. age-matched counterparts (37 significantly regulated protein). The latter appeared to indicate an upregulation in Sirtuin signaling and downregulation in oxidative phosphorylation, leukocyte extravasation, vascular endothelial growth factor (VEGF) signaling and integrin linked kinase (ILK) signaling. While the former also indicated an upregulation in Sirtuin signaling and downregulation in oxidative phosphorylation, but also a host of different pathways such as down regulation of the TCA cycle II, mammalian target of rapamycin (mTOR), hypoxia inducible factor 1α (HIF1α), Ciliary neurotrophic factor (CNTF), and N-Formylmethionyl-leucyl-phenylalanine (fMLP), interleukin 3 (IL3), chemokine receptor type 3 (CCR3), CXC chemokine receptor type 4 (CXCR4), thrombin and necroptosis signaling pathways. Thus suggesting an alteration in energy bioenergetic, oxidative damage, chemotactic and inflammatory signaling, BBB damage and cell death with increasing CAA severity.

With respect to the cell-type specific proteomic changes, we only noted significant changes in smooth muscle cell specific markers in AD cases with a low CAA score compared to their age- matched controls, possibly indicating an early involvement of arteriolar smooth muscle cells in CAA pathogenesis as amyloid deposits form around arteriole vessels. Significant changes in pericyte, smooth muscle cell, microglia, astrocyte and endothelial cell specific markers were observed in AD cases with a high CAA score compared to their age-matched controls, thus signifying the involvement of multiple cerebrovascular/perivascular cell types in the late degenerative stages of CAA.

To further delineate the differences between AD cases with low vs. high CAA scores, we also compared proteomic changes between their cerebrovessels and observed 84 significantly regulated proteins. Some of the main pathways identified involved downregulation of TCA cycle II, oxidative phosphorylation, mitochondrial dysfunction, and gluconeogenesis, further supporting the role of deficits in energy bioenergetics within cerebrovessels with CAA severity. With respect to changes in cell-type specific markers, we again revealed that smooth muscle cell specific markers played the greatest influence across the neuropathological staging of CAA in AD cases, followed by pericytes, and endothelial cells/astrocytes.

The role of the cerebrovasculature in driving AD pathogenesis has long been discussed. The vascular hypothesis was described a few decades ago to provide a vascular preclinical etiology for AD. Early evidence showed that cerebral perfusion, cortical blood flow and metabolic deficits were observed in MCI patients and those who go on to develop AD (De la Torre and Mussivand, 1993; Binnewijzend et al., 2016; Hays et al., 2016; de la Torre, 2018), beginning many years prior to the onset of their neurological symptoms. Seminal findings from the Nun dementia study (Snowdon, 1997) involving catholic sisters between 75 to 107yrs also demonstrated a prominent role for vascular lesions such as lacunar infarcts at autopsy, with these lesions serving as a protagonist for reducing the neuropathological threshold required for defining the staging of AD dementia (Nagy et al., 1997). Other vascular related morphological lesions have also been observed at autopsy in demented and non-demented elderly patients such as degeneration of small blood vessels, capillaries and perivascular end-feet processes (Miyakawa and Kuramoto, 1989; Hashimura et al., 1991; Kimura et al., 1991; Vinters et al., 1994; Kalaria, 1996; Farkas et al., 2000; Farkas and Luiten, 2001; Hauw et al., 2002; Zekry et al., 2002; Fernando and Ince, 2004; Østergaard et al., 2013); mitochondrial abnormalities and deposition of phagolysosomes and lipofuscin in cerebrovascular cells (Terry et al., 1964; Miyakawa and Kuramoto, 1989), blood brain barrier damage (Deane and Zlokovic, 2007) and immune cell extravasation (Itagaki et al., 1988; Rogers et al., 1988; Togo et al., 2002; Di Marco et al., 2015; Merlini et al., 2018). Moreover, vascular factors such as cardiovascular diseases, atherosclerosis, diabetes mellitus, hyperhomocysteinhemia, obesity and hypertension also increase the risk for AD in later life (Breteler, 2000; Kivipelto et al., 2001; Duron and Hanon, 2008; Ouldred and Bryant, 2010; de Bruijn and Ikram, 2014; O'Brien and Markus, 2014), alogether suggesting that vascular dysfunction may play a role in contributing to AD pathogenesis as we age. However, whether these vascular issues are a direct prelude to AD pathogenesis or a consequence remains elusive.

Vascular dysfunction manifested as brain hypoperfusion, hypoxia and hypometabolism are known among the modulators of cerebral amyloidogenesis and amyloid clearance (Mosconi, 2005; Zhang et al., 2007; Mawuenyega et al., 2010; Govindpani et al., 2019). In turn, deposition of amyloid-beta toxic species on vascular walls can also lead to changes typified by impairment in vascular hemodynamics and vessel rigidity leading to arterial and arteriole narrowing, increased cerebral blood pressure and further weakening of the vascular walls contributing to hypoperfusion and hypometabolism (Kalback et al., 2004; Tian et al., 2004). We partly observed this in our study in the form of changes to basement membrane proteins (implicating age-related arteriolosclerosis), activation of hypoxia inducible factor HIF1α, deficits in cerebrovessel energy bioenergetics and also from the strong correlation between CAA severity and the extent of proteomic changes in cerebrovessels. Our previous work has also confirmed a reduction in mural cell markers such as PDGFRβ and αSMA by ELISA method in the same batch of control and AD cerebrovessels used in this study (Ojo et al., 2021), signaling a possible degeneration of these cells in AD. These direct toxic effects of amyloid species have been confirmed in isolated cerebral tissue, in transgenic mouse models with CAA and cerebrovascular cell culture models after exposure to toxic amyloid species (Paris et al., 2003; Park et al., 2004; Tong et al., 2005; Nortley et al., 2019). As our proteomic study design focused on the end-stage analyses of cerebrovessels, we are unable to definitively determine whether the proteomic changes reported herein precede amyloid or tau pathologies, or are a consequence of these pathogenic protein species activating the molecular cascades of cerebrovascular events. Our study comparisons of young vs. aged control cases, and also AD cases stratified based on their CAA staging, does seem to point toward early cerebrovascular changes involving disturbances in energy metabolisms, oxidative stress and inflammation, which could have significant ramifications for brain and cerebrovascular physiology later in life.

To date, only few proteomic studies have been conducted to investigate brain vascular abnormalities in AD. Manousopoulou et al. (2017) used global quantitative proteomic analysis to interrogate endophenotypic profiles in large leptomeningeal arteries from patients with CAA (82.9 ± 7.5 yrs) compared to young (45.4 ± 3.9 yrs) and elderly controls (88.3 ± 8.6 yrs). Authors reported similar findings with our study demonstrating significant alterations in immune response and classical complement and extracellular matrix remodeling pathways in arteries affected by CAA compared to young and elderly controls. Clusterin (apolipoprotein J) and tissue inhibitor of metalloproteinases-3 (TIMP3) were the top significantly regulated proteins to be upregulated in CAA compared to young and elderly controls, and they were found to co-localize with amyloid-beta from CAA lesions. In our study, we also saw changes in clusterins in young vs. aged cerebrovessels, and early changes in matrix metalloproteinases such as MMP12 in CAA pathogenesis, suggesting a role for these proteins in driving the molecular damage in CAA pathogenesis.

Another study using laser dissection microscopy assisted quantitative mass spectrometry analysis was used to interrogate post-mortem human brain tissue of AD lesions with amyloid deposits in the brain parenchyma, predominant severe capillary CAA, and non-demented controls without amyloid deposits (Hondius et al., 2018). The authors identified 29 CAA-selective proteins. Notably increased levels of clusterin (CLU), apolipoprotein E (APOE) and serum amyloid P-component (APCS) were observed in AD brains with CAA, and collagen alpha-2(VI) (COL6A2) as highly selective markers unique to only CAA but absent in cases with parenchymal amyloid. Collagen makes up 50% of basement membrane proteins, and we also found increased levels of COL1A1 protein subunit in AD cases with low vs. high CAA scores, suggesting that extracellular remodeling is a key protective mechanisms against CAA pathogenesis, to combat weakening of vascular basement membrane integrity.

Animal studies have also been used to explore changes to the cerebrovascular proteome in AD pathogenesis. A prior study used gel-free and gel-based mass spectrometry to interrogate cerebrovascular proteome in 6.5 month old mice overexpressing APP compared to non-transgenic controls, and demonstrated over 190 proteins significantly regulated (Badhwar et al., 2017). The molecular changes identified included changes such as RNA/DNA damage, vascular cytoskeleton alterations, deregulation of the oxidoreductase system, oxidative stress, alterations in cerebrovascular vasocontractile tone, and vascular compliance, which were all in line with the outcomes reported in our current study. Some of these effects were rescued by treatment with pioglitazone, which acts through nuclear hormone receptor PPARγ to regulate lipid and glucose metabolism, mitochondrial bioenergetics, and inflammation. In our studies alterations in TCA cycle II, gluconeogenesis, sirtuin signaling, oxidative phosphorylation and mitochondrial dysfunction were observed in cerebrovessels with aging and AD pathogenesis, indicating that mitochondrial bioenergetics is a major event implicated in cerebrovascular dysfunction and diminished cerebral perfusion. Collectively these findings provide evidence for targeting energy metabolism and mitochondrial pathobiology in the cerebrovasculature as a potential therapeutic approach in the early stages of AD pathology.

We noted that AD cases with low or high CAA scores consisted of 40 or 67% APOE4 allele carriers, respectively, while only 20% of aged non-demented control cases were APOE4 allele carriers, suggesting that APOE4 genotype may be a driver of the events we reported in this study. APOE is thought to play a role in regulating the metabolism and perivascular drainage of Aβ and other soluble metabolites in extracellular fluids of the brain (Kim et al., 2009; Castellano et al., 2011; Cramer et al., 2012; Verghese et al., 2013; Kanekiyo et al., 2014). Early clues linking APOE with vascular degeneration was demonstrated in studies showing a link between APOE4 and increased toxic amyloid deposition around blood vessels in CAA (Greenberg et al., 1995; McCarron and Nicoll, 2000; Nelson et al., 2013; Rannikmäe et al., 2014). This has now been corroborated in transgenic models where an isoform specific (E4>E2 and/or E3) shift in Aβ from the brain parenchyma to arterioles was observed in the form of CAA and increased incidence of microhemorrhages (Greenberg et al., 1995; Holtzman et al., 2000; Fryer et al., 2003, 2005; Sullivan et al., 2008; Tai et al., 2011). APOE4 has also been shown to impact BBB permeability at the level of endothelial cells as demonstrated by an isoform specific (E4>E3) increase in the mRNA levels of influx Aβ transporters, RAGE (by seven-fold) (Donahue and Johanson, 2008). The APOE4 isoform has also been shown to negatively impact MMP9 induced (efflux transporter) LRP-1 shedding and its subsequent transport of APOE-Aβ complexes (Alonzo et al., 1998). Reductions in LRP1 levels were observed in this study, in corroboration with our previous work using the same batch of control and AD cerbrovessel samples and ELISA analyses (Alonzo et al., 1998).

Other studies have implicated a role for APOE4 in non-amyloidogenic mechanisms involving direct damage to the vasculature in AD. For example, a significant correlation between APOE4 allele and cerebral small-vascular diseases (CSVD) has been reported in cross-sectional studies involving AD and age-matched control cases (Utter et al., 2008; Schilling et al., 2013; Luo et al., 2017). APOE4 has also been linked with increased risk for fat or cholesterol build up in the middle cerebral artery in elderly patients (Kosunen et al., 1995; Zhang et al., 2018), and could lead to stenosis and the decrease in cerebral perfusion by smaller penetrating blood vessels. Moreover, CAA patients in the amyloid antibody trials who are APOE4 carriers typically show an increased propensity for microhemorrhages (Roher et al., 2011; Hanson et al., 2014; Salloway et al., 2014). Recent work has also confirmed the link between APOE4 and the accelerated breakdown of the BBB as an early event in AD (Zipser et al., 2007; Dickstein et al., 2010; Bell et al., 2012; Montagne et al., 2020). This appears to be driven by an APOE4 effect on the LRP1-Cyclophilin-NF-κB-MMP9 pathway in pericytes (Bell et al., 2012; Halliday et al., 2016; Montagne et al., 2020). Damage to mural cells could impact on the vasoconstrictive properties of cerebral microvessels leading to diminished blood flow and impaired cerebrovascular clearance (Hamilton, 2010; Khennouf et al., 2018; Nortley et al., 2019). While damage to the BBB can result in leukocyte extravasation and influx of blood borne proteins (such as fibrinogen and ferritin), which were both observed in this current study between control and AD cases.

One of the limitations of our study is the inability to detect the specific cellular origins of all the differentially expressed proteins in the cerebrovasculature, which constitutes a mixed cell population. Therefore, in future studies, we propose interrogating these omic changes at the single cell level using tools such as single cell RNAseq, RNAscope, single cell proteomic analyses and double-immunofluorescent staining. Another limitation of this work, is the bias in gender distribution across the different groups, with more females in the AD cases (60-70%) compared to the controls (~40%). Moreover, our young healthy control cohort coincidentally consisted of 60% APOE4 allele carriers, while the aged-matched control cases consisted of 20%. Thus our findings must be interpreted based on the bias across these groups as sex may serve as a biological variable driving our outcomes, while APOE4 genotype in the young cases may also mask some of the age-related effects observed in the study.

### Conclusion

We hypothesized that age-related and CAA dependent changes to the cerebrovascular proteome may explain the vascular contribution of aging and CAA in AD-related pathogenesis. We have observed that chemokine signaling, neurovascular signaling, hypoxia and oxidative damage and cell to cell signaling pathways were some of the prominent features of early age-related changes to cerebrovessels and this was driven by upstream regulators such as pro-survival and growth factor regulator RICTOR, transcriptional co-repressor TLE3, tau gene MAPT, amyloid precursor protein APP, and amyloidolytic enzyme subunit PSEN1.

We also revealed that proteomic changes to cerebrovessels across the neuropathological staging of CAA in AD brains appeared to be influenced early on by changes in energy bioenergetic, cellular homeostasis, BBB disturbances and immune cell infiltration. This was found to be driven by upstream regulators such as matrix metalloproteinase enzyme MMP12, endopeptidase enzyme CLPP, RICTOR and TLE3. The most common denominator in AD cerebrovessels from this study is the failure of mitochondrial function and energy bioenergetics. We hypothesize that mitochondrial failure in cerebrovessels may be a novel target in AD pathogenesis, and thus serve as a potential therapeutic target for rejuvenating cerebrovascular function and mitigating its negative consequences in AD.

It is worthy of note that low CAA groups represented cases with 50% high “NIA-R” AD diagnosis; while high CAA groups represented cases with 90%, suggesting that the effects observed are not absolutely driven by CAA score, but can be influenced by advanced amyloid plaque and NFT stage. Future studies exploring the differences in low and high CAA cases within a population with high NIA-R or ADNC diagnosis may help to determine the proteomic changes attributed primarily to CAA severity.

The findings nonetheless have provided a valuable molecular library of cerebrovascular pathobiological changes in aging and AD pathogenesis. The cerebrovasculature is crucial for maintaining neurovascular coupling and homeostatic equilibrium of the brain, providing adequate oxygen and glucose supply to cells to carry out their normal physiological functions. The proteomic changes we have identified may provide novel cerebrovascular based targets in developing therapeutic strategies to mitigate the damage to cerebrovessels early in the sequelae of AD pathogenesis. Our findings may also provide markers to aid in stratifying CAA patients during clinical trial studies for anti-amyloid immunotherapies that have an increased risk for microhemorrhages (Racke et al., 2005; Wilcock and Colton, 2009; Wilcock et al., 2011). Our future work will focus on interrogating and validating these novel targets and pathways and their functional significance in a larger cohorts of human samples and in controlled preclinical and *in vitro* studies.

## Data Availability Statement

The datasets presented in this study can be found in an online repository. The name of the repository and accession number can be found in the Methods section.

## Ethics Statement

Frozen human cortex tissue samples (from the inferior frontal gyrus) were provided mainly from Dr. Thomas Beach, Director of the Brain and Body Donation Program at Sun Health Research Institute (Sun City, AZ) in accordance with the institutional bioethics guidelines. Additional samples were requested from the NIH BrainBank repository (University of Maryland and Ican school of medicine, Mount Sinai, NY). The donors and their respective families provided hand written informed consent for autopsy and the subsequent use of brain tissue for research purposes. For this study, no further ethical approval was required as samples were obtained from deceased, de-identified, and consenting individuals.

## Disclosure

Dr. Bachmeier is a Research Scientist at the Bay Pines VA Healthcare System, Bay Pines, FL. Dr. Crawford is a Research Career Scientist at the James A. Haley Veterans Hospital, Tampa, FL. The content is solely the responsibility of the authors and does not necessarily represent the official views of the National Institutes of Health, the Department of Veterans Affairs, or the United States Government.

## Author Contributions

CB and JO conceived and directed the project. JO, GC, JR, and JE analyzed the results. JO, PV, BS, ME, and AE carried out the experiments. JO, CB, and FC drafted the manuscript. FC and MM were consultants on the project and provided their expertise and interpretation of the data. All authors contributed to the article and approved the submitted version.

## Conflict of Interest

JR was employed by company Boehringer Ingelheim Pharmaceuticals, Inc. The remaining authors declare that the research was conducted in the absence of any commercial or financial relationships that could be construed as a potential conflict of interest.

## Supplementary Material

Supplementary Figure 1 Network functions identified in young vs. aged control cerebrovascular tissue from the inferior frontal gyrus. Top Table shows associated network functions score identified from the ingenuity pathway analyses of significantly regulated proteins in young vs. aged controls. Network score was generated using IPA network generation algorithm that calculates the probability of finding the significantly regulated focus genes in a set of genes randomly selected from the relevant global molecular network. Network score cut off was set at >40. Schematic representation shows subcellular localization of proteins from each identified networks. Red—downregulated and Green—upregulated.

Supplementary Figure 2 Network functions identified in low CAA vs. aged-matched control cerebrovascular tissue from the inferior frontal gyrus. Top Table shows associated network functions score identified from the ingenuity pathway analyses of significantly regulated proteins in low CAA vs. aged-matched control cases. Network score was generated using IPA network generation algorithm that calculates the probability of finding the significantly regulated focus genes in a set of genes randomly selected from the relevant global molecular network. Network score cut off was set at >40. Schematic representation shows subcellular localization of proteins from each identified networks. Red—downregulated and Green—upregulated.

Supplementary Figure 3 Network functions identified in high CAA vs. aged-matched control cerebrovascular tissue from the inferior frontal gyrus. Top Table shows associated network functions score identified from the ingenuity pathway analyses of significantly regulated proteins in high CAA vs. aged-matched control cases. Network score was generated using IPA network generation algorithm that calculates the probability of finding the significantly regulated focus genes in a set of genes randomly selected from the relevant global molecular network. Network score cut off was set at >40. Schematic representation shows subcellular localization of proteins from each identified networks. Red—downregulated and Green—upregulated.

Supplementary Figure 4 Network functions identified in low vs. high CAA[AD] cerebrovascular tissue from the inferior frontal gyrus. Top Table shows associated network functions score identified from the ingenuity pathway analyses of significantly regulated proteins in low vs. high CAA [AD] cases. Network score was generated using IPA network generation algorithm that calculates the probability of finding the significantly regulated focus genes in a set of genes randomly selected from the relevant global molecular network. Network score cut off was set at >40. Schematic representation shows subcellular localization of proteins from each identified networks. Red—downregulated and Green—upregulated.

Supplementary Table 1 List of significantly regulated proteins in the cerebrovasculature of the inferior frontal gyrus of young vs. aged control cases. Data are expressed as the negative Log10 of the *p*-value (green horizontal bars–significance cut off set at >1.3), and the Log2 fold change between young vs. aged control cases. Heat map indicates downregulated (Red box) or upregulated (Blue box) proteins. Statistical analyses was performed using t-test after logarithmic transformation. *Red text highlights indicate 124 significantly proteins identified that were unique to only this comparison in our entire study*.

Supplementary Table 2 List of significantly regulated proteins in the cerebrovasculature of the inferior frontal gyrus of low CAA+ Alzheimer's disease and aged-matched control cases. Data are expressed as the negative Log10 of the *p*-value (green horizontal bars–significance cut off set at >1.3), and the Log2 fold change between low CAA [AD] vs. age-matched control cases. Heat map indicates downregulated (Red box) or upregulated (Blue box) proteins. Statistical analyses was performed using *t*-test after logarithmic transformation. *Red text highlights indicate 17 significantly proteins identified that were unique to only this comparison in our entire study*.

Supplementary Table 3 List of significantly regulated proteins in the cerebrovasculature of the inferior frontal gyrus in high CAA [AD] vs. age-matched control cases. Data are expressed as the negative Log10 of the *p*-value (green horizontal bars–significance cut off set at >1.3), and the Log2 fold change between high CAA [AD] vs. age-matched control cases. Heat map indicates downregulated (Red box) or upregulated (Blue box) proteins. Statistical analyses was performed using t-test after logarithmic transformation. *Red text highlights indicate 155 significantly proteins identified that were unique to only this comparison in our entire study*.

Supplementary Table 4 List of significantly regulated proteins in the cerebrovasculature of the inferior frontal gyrus in low vs. high CAA [AD] cases. Data are expressed as the negative Log10 of the p value (green horizontal bars–significance cut off set at >1.3), and the Log2 fold change between low vs. high CAA [AD] cases. Heat map indicates downregulated (Red box) or upregulated (Blue box) proteins. Statistical analyses was performed using t-test after logarithmic transformation. *Red text highlights indicate 36 significantly proteins identified that were unique to only this comparison in our entire study*.
