# Supplementary figures and images for: Molecular Pathobiology of the Cerebrovasculature in Aging and in Alzheimers Disease Cases With Cerebral Amyloid Angiopathy
Source: Front Aging Neurosci. 2021 May 17;13:658605. doi: 10.3389/fnagi.2021.658605 (PMC8166206; doi:10.3389/fnagi.2021.658605)

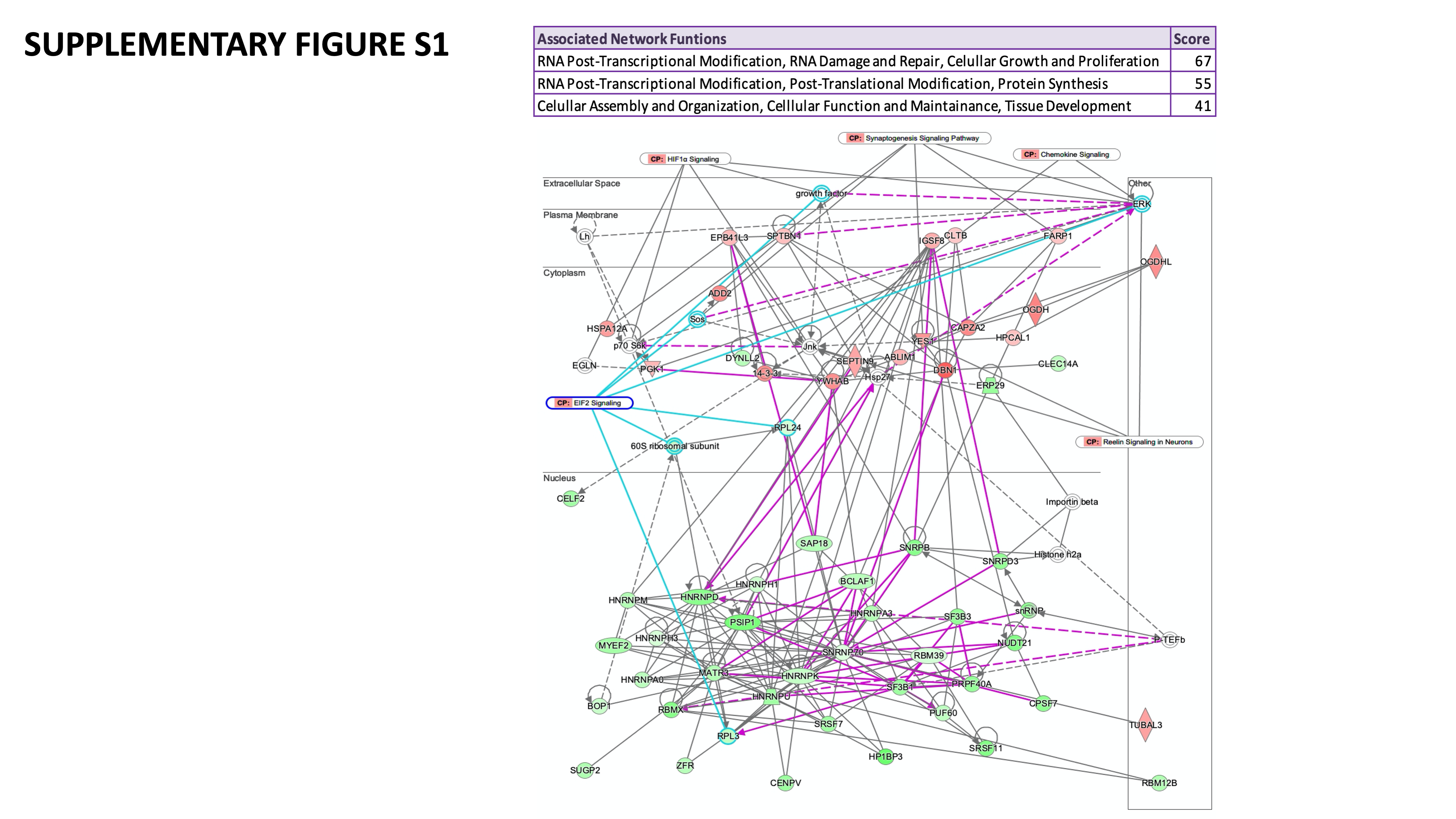

Supplement: Supplementary Figure 1 — Network functions identified in young vs. aged control cerebrovascular tissue from the inferior frontal gyrus. Top Table shows associated network functions score identified from the ingenuity pathway analyses of significantly regulated proteins in young vs. aged controls. Network score was generated using IPA network generation algorithm that calculates the probability of finding the significantly regulated focus genes in a set of genes randomly selected from the relevant global molecular network. Network score cut off was set at >40. Schematic representation shows subcellular localization of proteins from each identified networks. Red—downregulated and Green—upregulated. [file Image_5.TIFF]

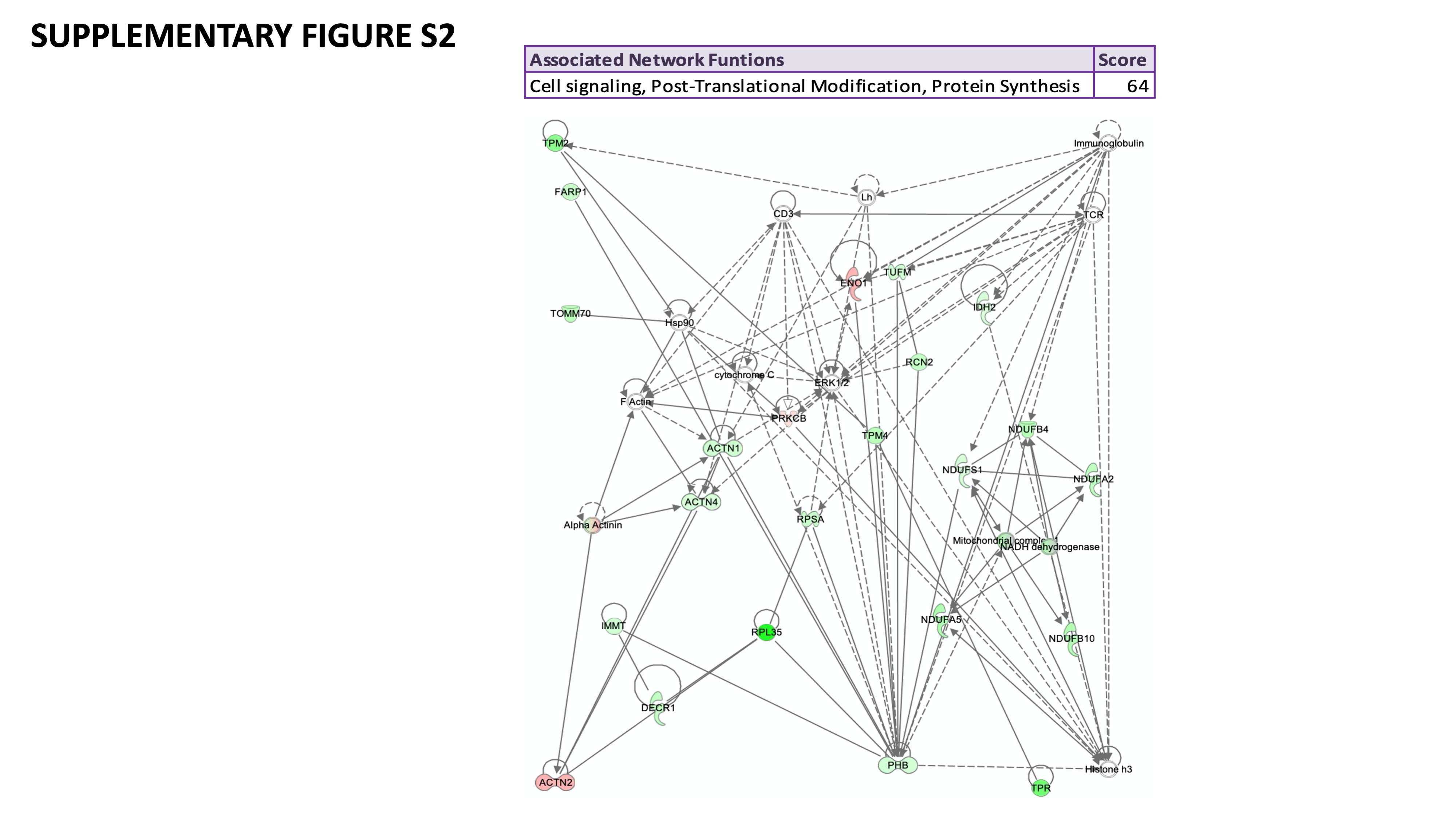

Supplement: Supplementary Figure 2 — Network functions identified in low CAA vs. aged-matched control cerebrovascular tissue from the inferior frontal gyrus. Top Table shows associated network functions score identified from the ingenuity pathway analyses of significantly regulated proteins in low CAA vs. aged-matched control cases. Network score was generated using IPA network generation algorithm that calculates the probability of finding the significantly regulated focus genes in a set of genes randomly selected from the relevant global molecular network. Network score cut off was set at >40. Schematic representation shows subcellular localization of proteins from each identified networks. Red—downregulated and Green—upregulated. [file Image_6.TIFF]

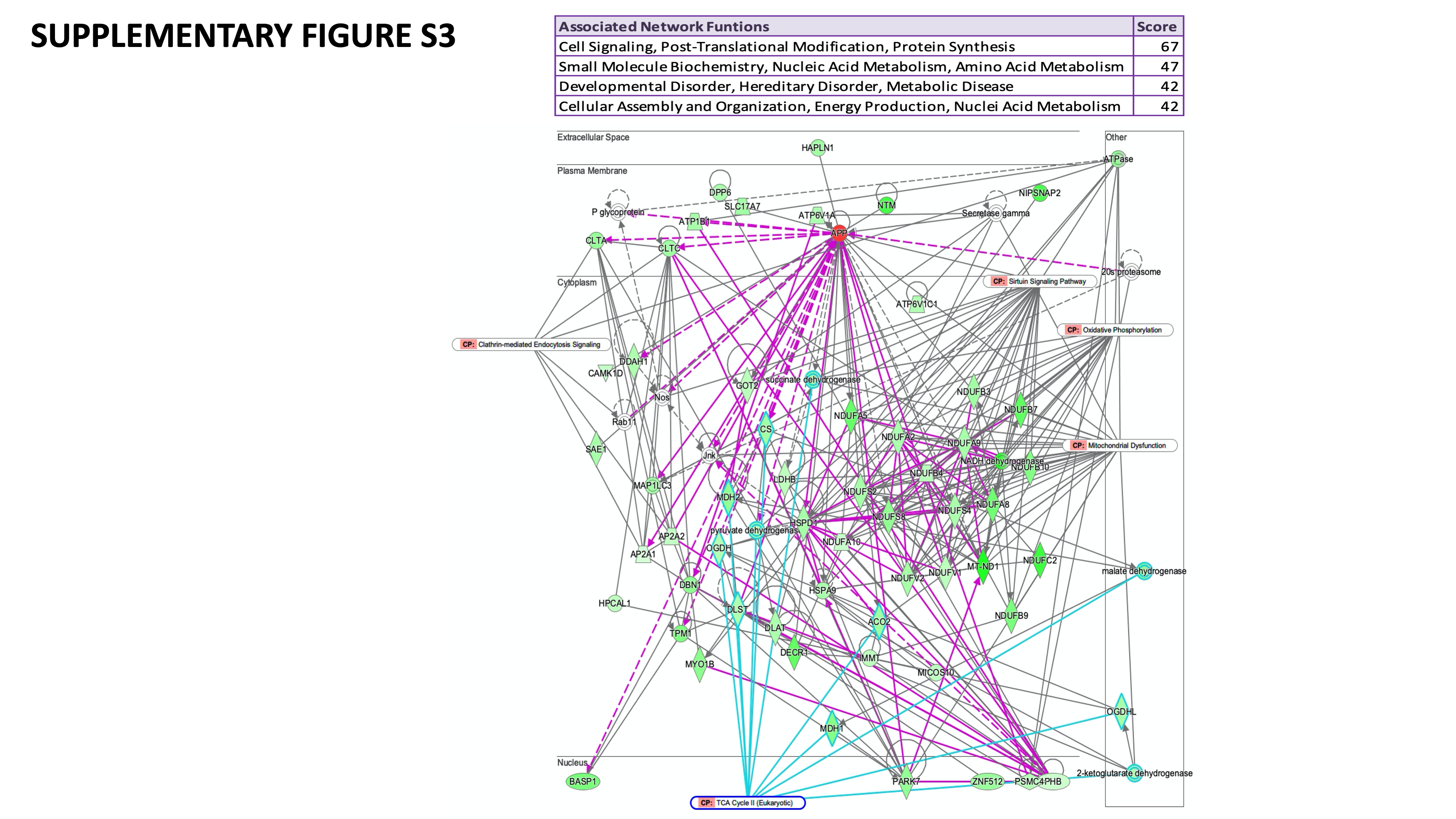

Supplement: Supplementary Figure 3 — Network functions identified in high CAA vs. aged-matched control cerebrovascular tissue from the inferior frontal gyrus. Top Table shows associated network functions score identified from the ingenuity pathway analyses of significantly regulated proteins in high CAA vs. aged-matched control cases. Network score was generated using IPA network generation algorithm that calculates the probability of finding the significantly regulated focus genes in a set of genes randomly selected from the relevant global molecular network. Network score cut off was set at >40. Schematic representation shows subcellular localization of proteins from each identified networks. Red—downregulated and Green—upregulated. [file Image_7.TIFF]

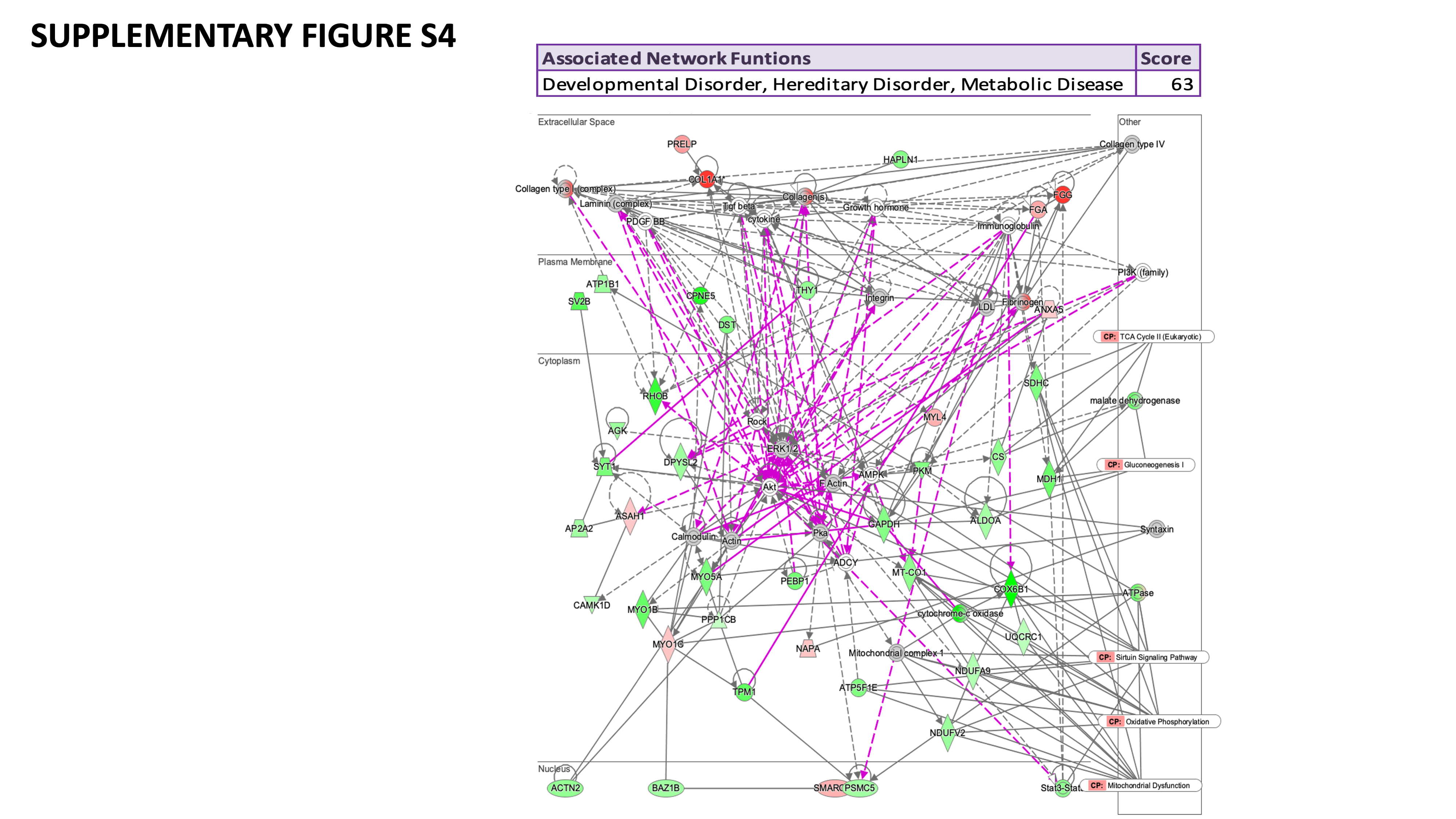

Supplement: Supplementary Figure 4 — Network functions identified in low vs. high CAA[AD] cerebrovascular tissue from the inferior frontal gyrus. Top Table shows associated network functions score identified from the ingenuity pathway analyses of significantly regulated proteins in low vs. high CAA [AD] cases. Network score was generated using IPA network generation algorithm that calculates the probability of finding the significantly regulated focus genes in a set of genes randomly selected from the relevant global molecular network. Network score cut off was set at >40. Schematic representation shows subcellular localization of proteins from each identified networks. Red—downregulated and Green—upregulated. [file Image_8.TIFF]

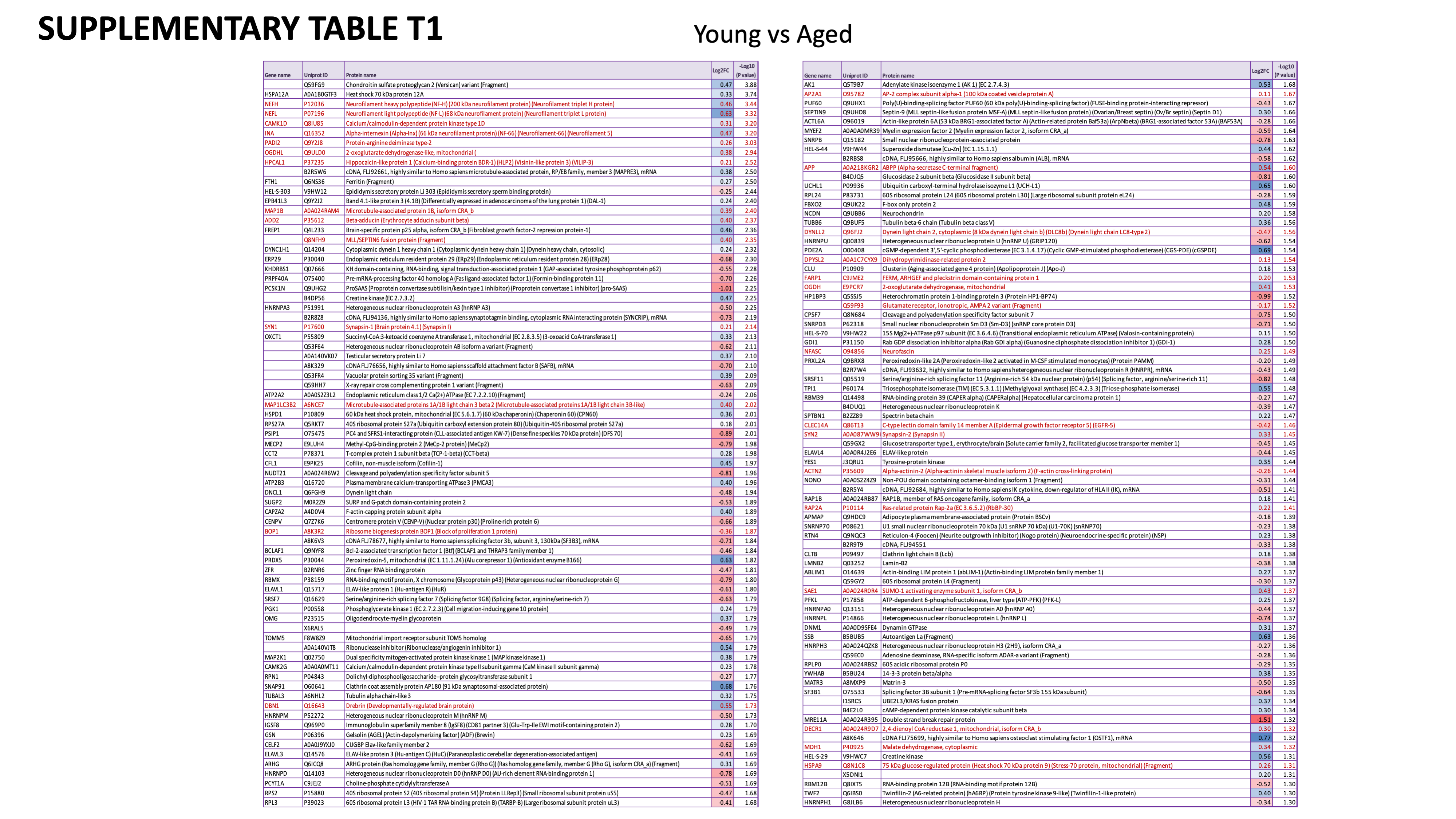

Supplement: Supplementary Table 1 — List of significantly regulated proteins in the cerebrovasculature of the inferior frontal gyrus of young vs. aged control cases. Data are expressed as the negative Log10 of the p-value (green horizontal bars–significance cut off set at >1.3), and the Log2 fold change between young vs. aged control cases. Heat map indicates downregulated (Red box) or upregulated (Blue box) proteins. Statistical analyses was performed using t-test after logarithmic transformation. Red text highlights indicate 124 significantly proteins identified that were unique to only this comparison in our entire study. [file Image_1.TIFF]

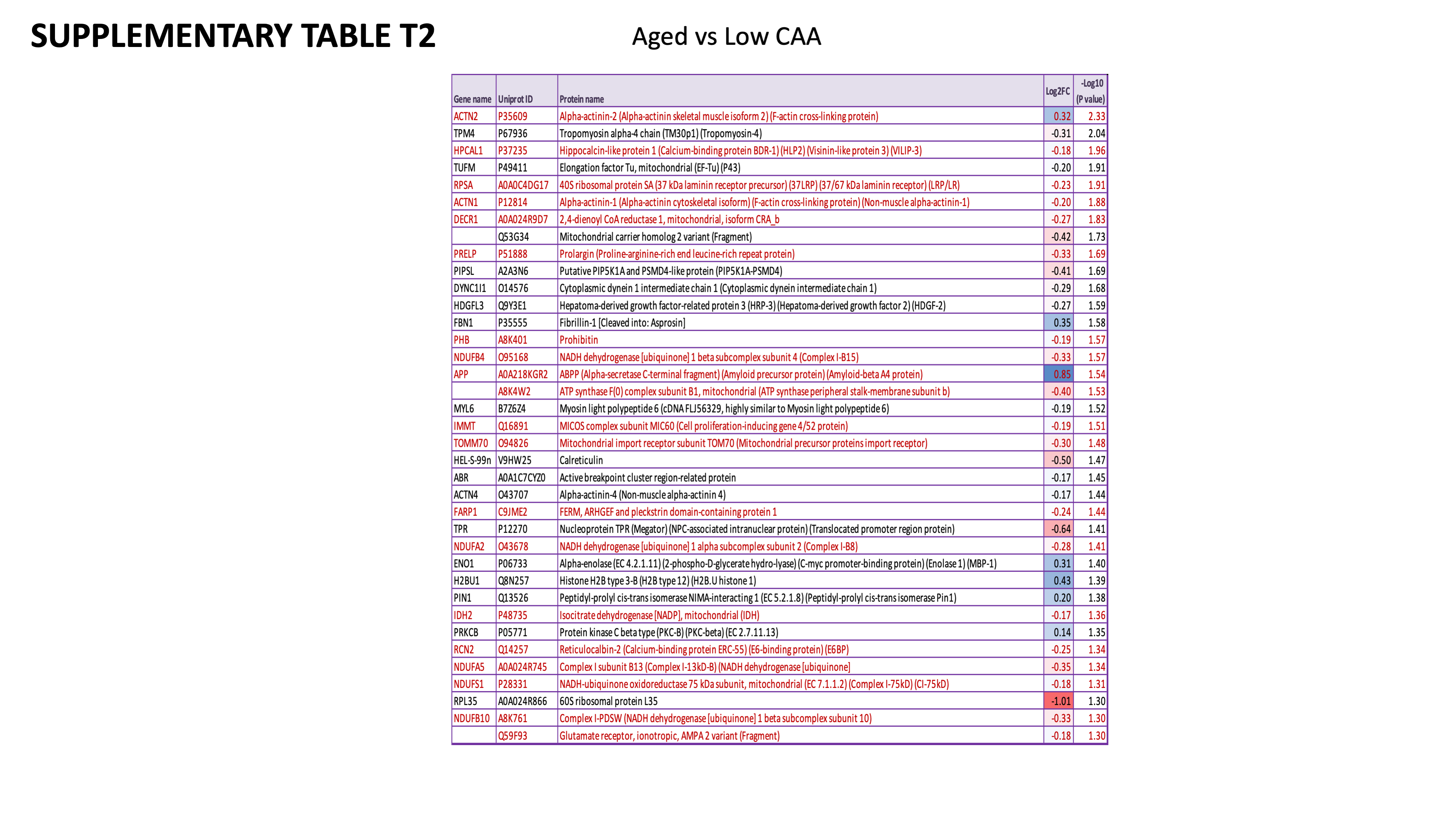

Supplement: Supplementary Table 2 — List of significantly regulated proteins in the cerebrovasculature of the inferior frontal gyrus of low CAA+ Alzheimer's disease and aged-matched control cases. Data are expressed as the negative Log10 of the p-value (green horizontal bars–significance cut off set at >1.3), and the Log2 fold change between low CAA [AD] vs. age-matched control cases. Heat map indicates downregulated (Red box) or upregulated (Blue box) proteins. Statistical analyses was performed using t-test after logarithmic transformation. Red text highlights indicate 17 significantly proteins identified that were unique to only this comparison in our entire study. [file Image_2.TIFF]

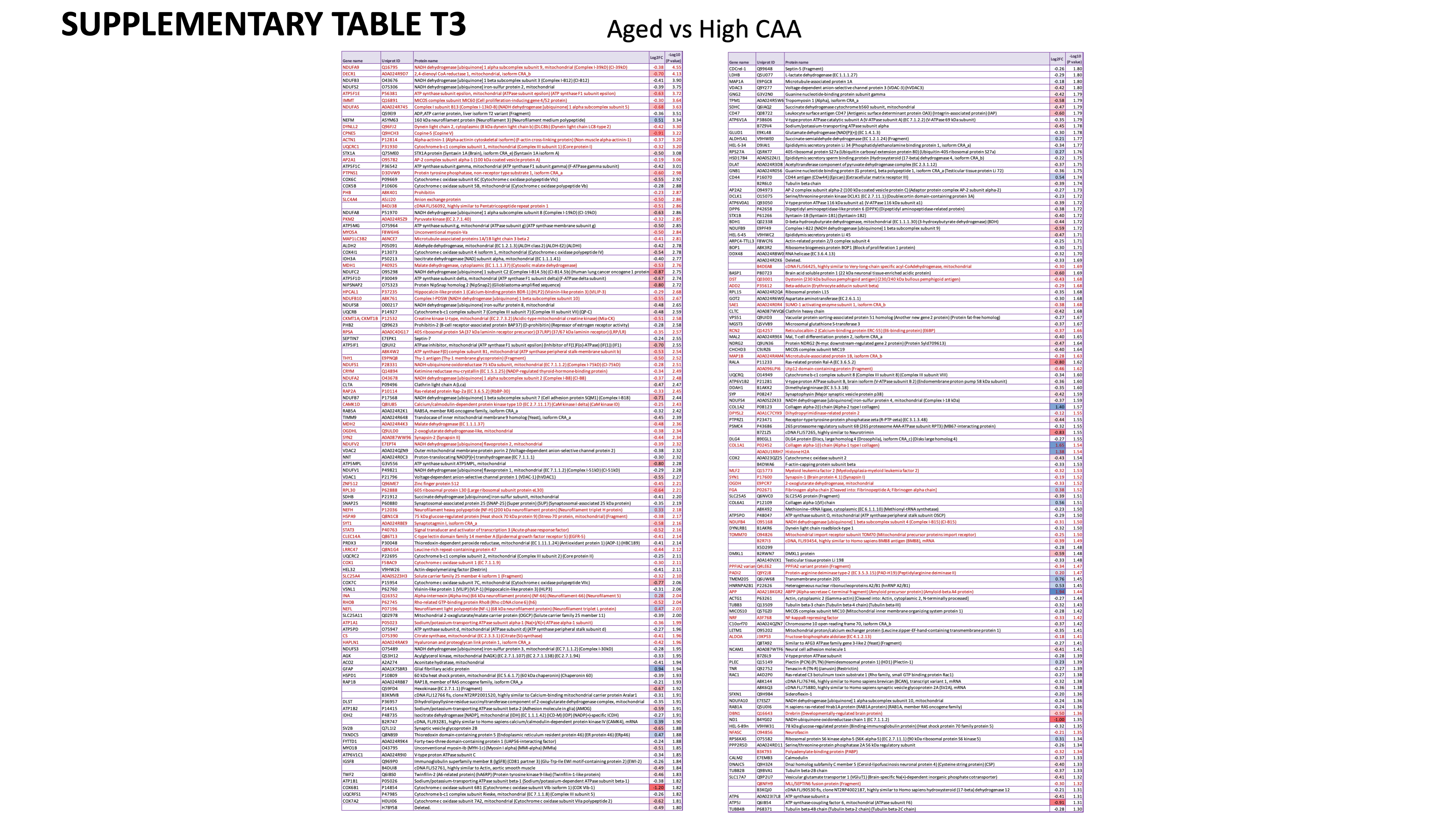

Supplement: Supplementary Table 3 — List of significantly regulated proteins in the cerebrovasculature of the inferior frontal gyrus in high CAA [AD] vs. age-matched control cases. Data are expressed as the negative Log10 of the p-value (green horizontal bars–significance cut off set at >1.3), and the Log2 fold change between high CAA [AD] vs. age-matched control cases. Heat map indicates downregulated (Red box) or upregulated (Blue box) proteins. Statistical analyses was performed using t-test after logarithmic transformation. Red text highlights indicate 155 significantly proteins identified that were unique to only this comparison in our entire study. [file Image_3.TIFF]

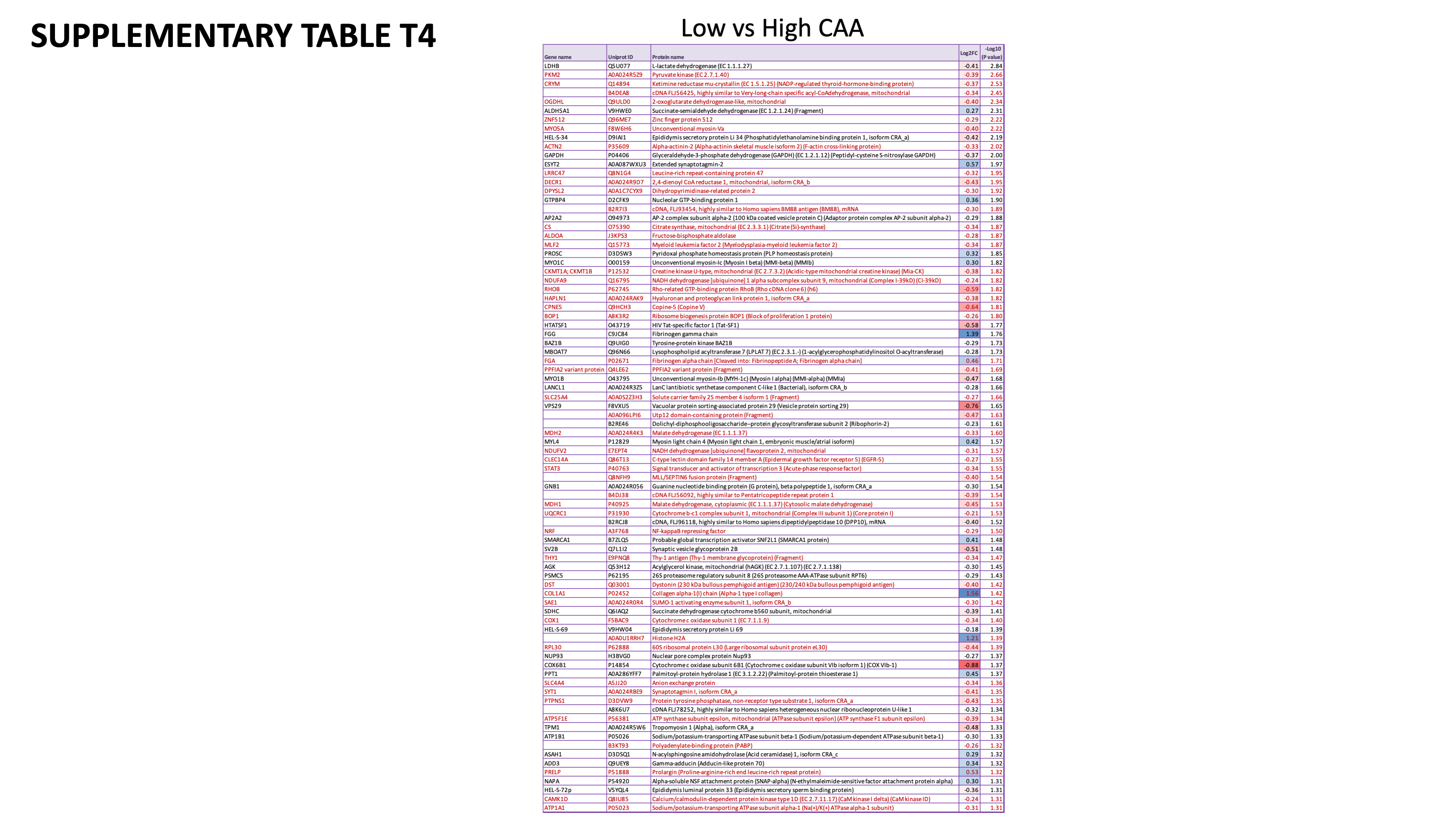

Supplement: Supplementary Table 4 — List of significantly regulated proteins in the cerebrovasculature of the inferior frontal gyrus in low vs. high CAA [AD] cases. Data are expressed as the negative Log10 of the p value (green horizontal bars–significance cut off set at >1.3), and the Log2 fold change between low vs. high CAA [AD] cases. Heat map indicates downregulated (Red box) or upregulated (Blue box) proteins. Statistical analyses was performed using t-test after logarithmic transformation. Red text highlights indicate 36 significantly proteins identified that were unique to only this comparison in our entire study. [file Image_4.TIFF]
